# Costs and cost-effectiveness of influenza illness and vaccination in low- and middle-income countries: A systematic review from 2012 to 2022

**DOI:** 10.1371/journal.pmed.1004333

**Published:** 2024-01-05

**Authors:** Radhika Gharpure, Anna N. Chard, Maria Cabrera Escobar, Weigong Zhou, Molly M. Valleau, Tat S. Yau, Joseph S. Bresee, Eduardo Azziz-Baumgartner, Sarah W. Pallas, Kathryn E. Lafond

**Affiliations:** 1 U.S. Centers for Disease Control and Prevention, Atlanta, Georgia, United States of America; 2 Task Force for Global Health, Atlanta, Georgia, United States of America; Boston University School of Public Health, UNITED STATES

## Abstract

**Background:**

Historically, lack of data on cost-effectiveness of influenza vaccination has been identified as a barrier to vaccine use in low- and middle-income countries. We conducted a systematic review of economic evaluations describing (1) costs of influenza illness; (2) costs of influenza vaccination programs; and (3) vaccination cost-effectiveness from low- and middle-income countries to assess if gaps persist that could hinder global implementation of influenza vaccination programs.

**Methods and findings:**

We performed a systematic search in Medline, Embase, Cochrane Library, CINAHL, and Scopus in January 2022 and October 2023 using a combination of the following key words: “influenza” AND “cost” OR “economic.” The search included studies with publication years 2012 through 2022. Studies were eligible if they (1) presented original, peer-reviewed findings on cost of illness, cost of vaccination program, or cost-effectiveness of vaccination for seasonal influenza; and (2) included data for at least 1 low- or middle-income country. We abstracted general study characteristics and data specific to each of the 3 study types. Of 54 included studies, 26 presented data on cost-effectiveness, 24 on cost-of-illness, and 5 on program costs. Represented countries were classified as upper-middle income (UMIC; *n* = 12), lower-middle income (LMIC; *n* = 7), and low-income (LIC; *n* = 3). The most evaluated target groups were children (*n* = 26 studies), older adults (*n* = 17), and persons with chronic medical conditions (*n* = 12); fewer studies evaluated pregnant persons (*n* = 9), healthcare workers (*n* = 5), and persons in congregate living settings (*n* = 1). Costs-of-illness were generally higher in UMICs than in LMICs/LICs; however, the highest national economic burden, as a percent of gross domestic product and national health expenditure, was reported from an LIC. Among studies that evaluated the cost-effectiveness of influenza vaccine introduction, most (88%) interpreted at least 1 scenario per target group as either cost-effective or cost-saving, based on thresholds designated in the study. Key limitations of this work included (1) heterogeneity across included studies; (2) restrictiveness of the inclusion criteria used; and (3) potential for missed influenza burden from use of sentinel surveillance systems.

**Conclusions:**

The 54 studies identified in this review suggest an increased momentum to generate economic evidence about influenza illness and vaccination from low- and middle-income countries during 2012 to 2022. However, given that we observed substantial heterogeneity, continued evaluation of the economic burden of influenza illness and costs/cost-effectiveness of influenza vaccination, particularly in LICs and among underrepresented target groups (e.g., healthcare workers and pregnant persons), is needed. Use of standardized methodology could facilitate pooling across settings and knowledge sharing to strengthen global influenza vaccination programs.

## Abstract 2

In a systematic review of economic evaluations, Gharpure and team examine the costs of influenza illness and vaccination programs in low- and middle-income countries to assess potential barriers to global implementation. Full length: In this systematic review of economic evaluations, Radhika Gharpure and colleagues examine the costs of influenza illness, the costs of influenza vaccination programs, and the cost-effectiveness of vaccination from low- and middle-income countries to assess whether gaps remain that could hinder global implementation of influenza vaccination programs.

## Author summary

**Why was this study done?:**

- Cost-effectiveness analyses and other economic evaluations can provide important information to guide evidence-based decision-making, resource allocation, and long-term investment in vaccination by demonstrating value-for-money.
- Cost-effectiveness analyses require accurate input data, including the costs of influenza illness, costs of vaccination, and impact of the vaccination program, to yield relevant and reliable results.

**What did the researchers do and find?:**

- We conducted a systematic review of studies describing the costs of influenza illness, costs of influenza vaccination programs, and influenza vaccination cost-effectiveness from low- and middle-income country settings during 2012 to 2022, given the availability of updated global tools for economic evaluations.
- We collated the data from these articles, by study type and vaccination target group, to identify remaining gaps.
- We identified 54 eligible studies published during 2012 to 2022, representing an increase from prior years, but studies from low-income countries and for specific target groups such as pregnant persons and healthcare workers were limited.

**What do these findings mean?:**

- Additional studies from low-income countries and underrepresented target groups would strengthen the evidence regarding value-for-money, as robust, global economic data are critical to design and maintain sustainable influenza vaccination programs.
- Standardization of methodology across future economic evaluations, including considerations to capture the full spectrum of influenza-associated illness, could allow for pooled estimates and meta-analyses.
- The main limitations of this review were the variability across studies, limiting our ability to generalize and compare findings, as well as restrictiveness of the inclusion criteria and potential for missed influenza burden by sentinel surveillance.

## Introduction

Seasonal influenza vaccination is a key intervention to prevent morbidity and mortality from influenza virus infections. The World Health Organization (WHO) Strategic Advisory Group of Experts on Immunization (SAGE) recommends that countries starting or expanding influenza vaccination programs prioritize specific target groups at high risk for transmission or severe disease, including healthcare workers, individuals with chronic medical conditions, older adults, and pregnant persons [1]. Additionally, depending on priorities, available resources, and feasibility, countries might consider additional target groups for vaccination, including young children, persons in congregate living settings, systematically disadvantaged populations, and indigenous populations [1]. As of 2018, 118 of 194 (61%) WHO member states had an influenza vaccination policy [2], while low- and middle-income countries represent 40% of the world’s population and have a high burden of influenza illness [3–5]; they constituted 85% of countries without a policy [2].

A 2019 survey indicated that lack of data on cost-effectiveness of influenza vaccination programs was a key barrier to initiating and expanding influenza vaccination programs in low- and middle-income countries [6]. Cost-effectiveness analyses and other economic evaluations can provide important information to guide evidence-based decision-making, resource allocation, and long-term investment in vaccination by demonstrating value-for-money; however, these evaluations require accurate input data, including the costs of influenza illness, costs of vaccination, and impact of the vaccination program, in order to yield relevant and reliable results [7].

To help countries better assess the value of influenza vaccination, the WHO and partners have developed standardized tools and updated guidance in recent years for economic evaluations regarding influenza illness and vaccination. These include 2016 guidance on estimating influenza economic burden [8,9], 2016 guidance on economic evaluations for influenza vaccination, including cost-effectiveness analyses [10,11], and a 2020 update to the Seasonal Influenza Immunization Costing Tool (SIICT) [12]. While previous systematic reviews have described economic data for influenza from low- and middle-income countries [13–17], these were generally conducted prior to the availability of these tools; more recent reviews have described data from high-income settings [16,18,19], focused on specific target groups [20–22], or addressed questions such as the comparative cost-effectiveness of quadrivalent and trivalent vaccines [23]. To summarize recent data and assess remaining gaps, we conducted an updated systematic review of studies describing the costs of influenza illness, costs of influenza vaccination programs, and influenza vaccination cost-effectiveness from low- and middle-income country settings published during 2012 to 2022.

## Methods

This review followed the Preferred Reporting Items for Systematic Review and Meta-analysis (PRISMA) guidelines for systematic reviews (S1 PRISMA Checklist) and was registered at PROSPERO (international prospective register of systematic reviews) under protocol number CRD42022304803.

### Search strategy and study selection

We performed a systematic search using Medline, Embase, Cochrane Library, CINAHL, and Scopus in January 2022 for studies with a publication year of 2012 through 2021. The search was updated in October 2023 to include studies published in 2022. Search terms were a combination of the following key words: “influenza” AND “cost” OR “economic;” specific search syntax for each database is provided in **S1 Table**.

Studies were eligible if they met the following inclusion criteria: (1) presented original, peer-reviewed findings on at least one of the following: (a) cost of illness, (b) cost of vaccination program, or (c) cost-effectiveness, cost-utility, or cost-benefit of vaccination (hereafter referred to as “cost-effectiveness studies”) for seasonal influenza; and (2) included data for at least 1 low- or middle-income country based on World Bank income group classification during the study period of each publication [24]. We excluded studies that: (1) did not present original or peer-reviewed findings (e.g., literature reviews, conference abstracts, and editorials); (2) only presented data about infection with or vaccination for pandemic or novel influenza viruses (e.g., influenza A(H1N1)2009 pandemic strain), as these were not considered representative of seasonal influenza infection and/or vaccination; (3) included data from mid-2009 through mid-2010 that could not be disaggregated from other results, as these months were considered to represent the global influenza A(H1N1)2009 pandemic period [25]; or (4) presented data only from March 2020 through the end of 2022, as these years represented the global Coronavirus Disease 2019 (COVID-19) pandemic. Studies in any language were eligible for inclusion.

Specifically, cost-of-illness studies were required to use a case definition of laboratory-confirmed influenza (LCI) or syndromic definitions of influenza-like-illness (ILI) and/or severe acute respiratory infection (SARI), though estimates could then be extrapolated to include other disease presentations (e.g., non-medically attended illnesses). Program cost studies were required to present the monetary value of resources required for an influenza vaccination program; studies that described only the cost of vaccine purchase were excluded. Cost-effectiveness studies were required to include a comparison of influenza vaccination versus no vaccination or modifications to current vaccination program (e.g., increase in vaccination coverage); studies that only compared the cost-effectiveness of different influenza vaccine products (e.g., quadrivalent versus trivalent, adjuvanted versus non-adjuvanted, or live attenuated versus inactivated) were not included.

Titles and abstracts identified by the search strategy were independently screened by 2 reviewers (RG, ANC, MCE, WZ, or KEL) for eligibility; a publication was included for full-text review if either reviewer flagged it as potentially eligible. English-language full texts were again reviewed by 2 reviewers (RG, ANC, MCE, WZ, or MMV) for eligibility, with a third reviewer resolving any conflicting decisions. Identified publications in other languages were reviewed by a single native-language speaker (Mandarin Chinese, Russian, Spanish, and Bulgarian). All screening procedures were performed using Covidence, a web-based collaboration software platform for systematic reviews [26]. We also reviewed references from included studies to identify additional relevant literature for inclusion.

### Data extraction and quality assessment

Data from English-language publications were independently extracted by 2 reviewers (RG, ANC, MCE, WZ, or MMV), and disagreement was resolved by a discussion between the reviewers and consultation with a third reviewer if necessary. Data from publications in Mandarin Chinese were abstracted by a single native-language speaker (WZ or TSY); no other non-English publications met inclusion criteria.

A standardized Microsoft Excel-based data extraction form was developed to include the following information for all studies: country, study period, study methods, SAGE target group(s) represented, economic evaluation perspective, and funding source. Additionally, for cost-of-illness studies, we abstracted direct and indirect costs of outpatient visits and hospitalizations, as well as national economic burden if reported. For program cost studies, we abstracted financial and economic costs both including and excluding vaccine procurement. Financial costs were incremental monetary expenditures made for the influenza vaccination program; economic costs included all financial costs as well as the value of existing resources and donations (as categorized by study authors). For cost-effectiveness studies, we abstracted the study intervention(s), comparator(s), incremental cost-effectiveness ratio (ICER), ICER interpretation, and cost-effectiveness threshold. If reported, we preferentially abstracted median values for economic variables; if medians were not reported, we abstracted mean values or ranges. We did not contact study authors to request additional data.

We used World Bank data to classify the income group of countries during the study period [24]; if countries changed income group classification during the study period for a single publication, the higher classification was used. If multiple studies were published before and after a change in income group classification, the country was classified into multiple income groups corresponding to the classification during each study period. Additionally, we used World Bank data to obtain the gross domestic product (GDP) of countries during the study period [24]; for multiyear studies, the final year of the study period was used. For cost-of-illness and program cost studies, we also used WHO data to obtain the Current Health Expenditure and Domestic General Government Health Expenditure, respectively, of countries during the study period [27]. If no study period was specified, we used 3 years prior to the publication year for all relevant inputs as in prior systematic reviews [23].

For each English-language publication, 2 reviewers (RG, ANC, MCE, WZ, or MMV) assessed study quality and risk of bias using the Consolidated Health Economic Evaluation Reporting Standard (CHEERS) checklist [28]; for non-English publications, 1 native speaker (WZ or TSY) completed the CHEERS checklist. The checklist includes 24 criteria developed to ensure standardized reporting across economic studies; all 24 were assessed for cost-effectiveness studies, and modified sets of 13 and 15 criteria were used for cost-of-illness and program cost studies, respectively (**S2 Table**).

### Data conversion and analysis

We converted all currencies to US dollars (US$) using the International Monetary Fund official exchange rate for the nominal year [24] and then inflated all results to 2022 US$ using the US Bureau of Economic Analysis GDP implicit price deflator [29,30]. If a nominal currency year was not presented in the study, we used the final year of the study period or, if the study period was not stated, 3 years prior to the publication year. We calculated the gross national economic burden and program cost, when reported, as a proportion of the national GDP and the national health expenditure. Additionally, we collated direct and indirect costs by SAGE target group and income group and reported ranges across strata. Similarly, we also collated ICER results by SAGE target group and income group and calculated the proportion of studies that interpreted findings as “cost-saving” (ICER<0), “cost-effective” (dependent on cost-effectiveness threshold specified in the study), or “not cost-effective.” All analyses were performed using SAS (version 9.4) and Microsoft Excel.

### Patient and public involvement

As this was a systematic review of published literature, patients and the public were not involved in the design, conduct, reporting, or dissemination plans of this work.

## Results

### Study characteristics and quality assessment

**Fig 1:**
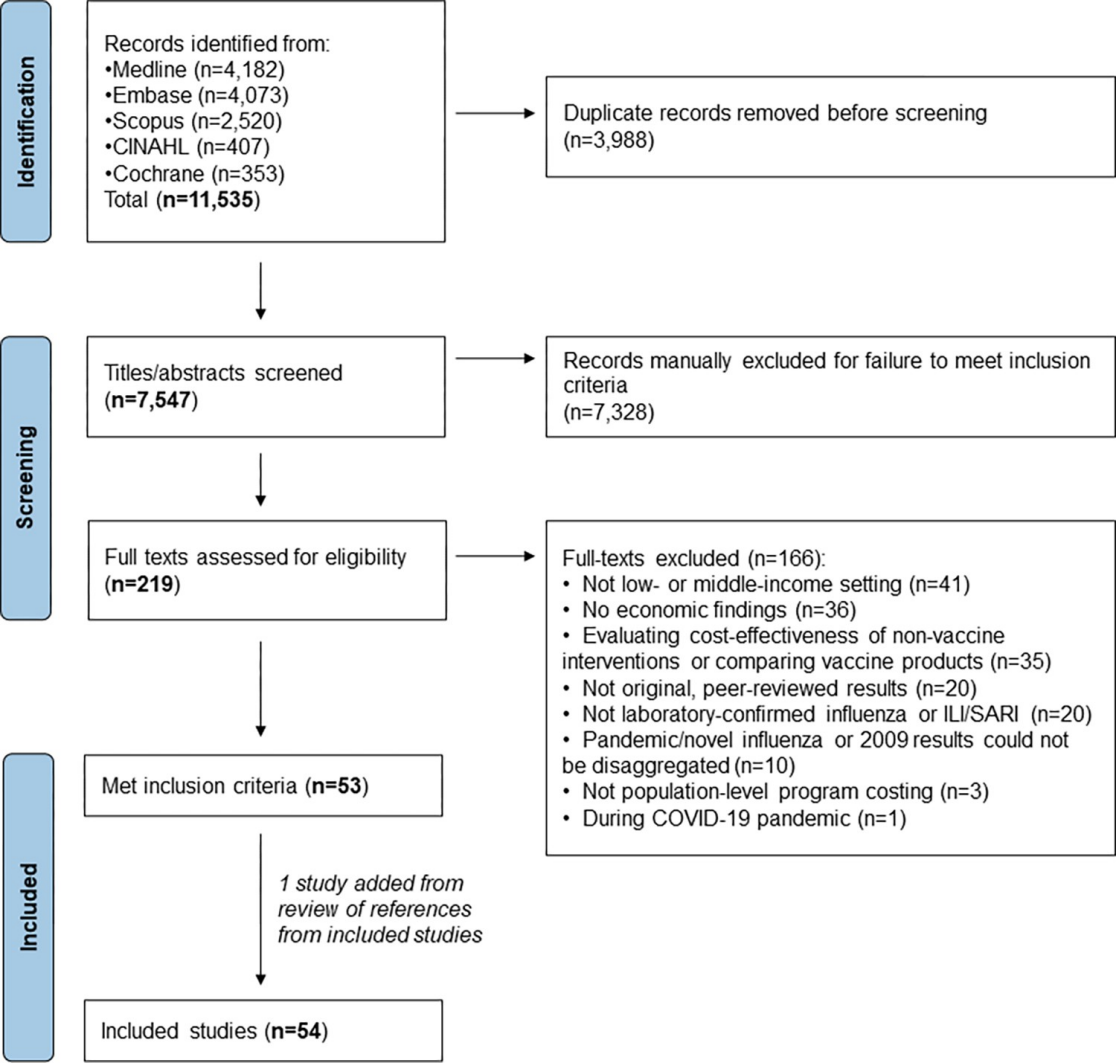
PRISMA flow diagram of study selection process. ILI, influenza-like illness; SARI, severe acute respiratory infection.

Of 7,547 total studies identified, 54 met eligibility criteria and were included in this review, including 46 English-language and 8 Chinese-language studies (**Fig 1 and S3 Table**). Study characteristics are presented in **Table 1**; a total of 26 studies presented cost-effectiveness findings, 24 presented cost-of-illness, and 5 presented program costs. Studies included data from 21 country settings, which were classified as upper-middle income countries (UMICs; *n* = 12), lower-middle income countries (LMICs; *n* = 7), and low-income countries (LICs; *n* = 3); 1 country, China, was classified as both UMIC and LMIC corresponding to multiple studies before and after an upward change in World Bank classification in 2010. These 21 countries represented 13% of 157 countries/territories classified as low- or middle-income countries in any year during 2005 (earliest year of data presented in included studies) through 2022. The most frequently evaluated SAGE target groups were children (*n* = 26 studies, inclusive of children aged <18 years), older adults (*n* = 17, inclusive of adults aged ≥60 years), and persons with chronic medical conditions (*n* = 12); fewer studies evaluated pregnant persons (*n* = 9), healthcare workers (*n* = 5), and persons in congregate living settings (*n* = 1).

**Table 1:** Number of included studies by income group classification, region, Strategic Advisory Committee of Experts on Immunization (SAGE) target group, and study type.

Income group and region	Total no. studies included	Total no. countries represented	No. studies by SAGE target group^1^							No. studies by study type		
			None (general population)	Children^2^	Older adults	Persons with chronic medical conditions	Pregnant persons	Healthcare workers	Persons in congregate living settings	Cost-of-illness	Cost-of-program	Cost-effectiveness
**Total**	**54** ^3^	**21** ^4^	**13**	**26**	**17**	**12**	**9**	**5**	**1**	**24** ^5^	**5**	**26** ^5^
**UMICs**	**44**	**12**	**7**	**22**	**14**	**11**	**6**	**3**	**1**	**18**	**4**	**22**
East Asia and Pacific	25	3	2	13	9	6	2	2	1	10	2	13
Latin America and Caribbean	9	4	3	7	0	1	0	0	0	4	0	5
Sub-Saharan Africa	6	1	2	2	3	3	4	0	0	2	1	3
Europe and Central Asia	4	4	0	0	2	1	0	1	0	2	1	1
**LMICs**	**11**	**7**	**5**	**4**	**3**	**1**	**1**	**2**	**0**	**7**	**0**	**4**
East Asia and Pacific	5	3	4	1	2	0	1	1	0	3	0	2
Sub-Saharan Africa	2	1	1	2	0	0	0	0	0	1	0	1
Europe and Central Asia	2	1	0	0	1	0	0	1	0	1	0	1
Latin America and Caribbean	1	1	0	1	0	0	0	0	0	1	0	0
South Asia	1	1	0	0	0	1	0	0	0	1	0	0
**LICs**	**3**	**3**	**1**	**0**	**0**	**0**	**2**	**0**	**0**	**2**	**1**	**1**
Sub-Saharan Africa	2	2	0	0	0	0	2	0	0	1	1	1
South Asia	1	1	1	0	0	0	0	0	0	1	0	0

^1^Vaccination target groups as defined in WHO SAGE guidance [1]. Several studies reported data on >1 target group.

^2^Although SAGE recommendations specifically reference children aged <5 years [1], publications with data for children aged <18 years were included.

^3^Three studies [42,44,73] included countries from multiple income groups and/or regions.

^4^One country (China) changed income classification from LMIC to UMIC in 2010 and was counted in both groups corresponding to studies assessing time periods before and after this year.

^5^One study [54] reported original data for both cost-of-illness and cost-effectiveness.

LIC, low-income country; LMIC, lower-middle income country; UMIC, upper-middle income country; WHO, World Health Organization.

Quality assessment scores indicated that the quality of included studies was acceptable; median scores by study type were 12 out of 13 (92%; interquartile range [IQR] 92% to 100%) for cost-of-illness, 14 out of 15 (93%; IQR 93% to 100%) for program costs, and 23 out of 24 (96%; IQR 84% to 100%) for cost-effectiveness studies (**S1 Fig**); only 3 of all 54 studies (6%) scored <75%. Of 48 studies that reported a funding source, 8 (18%) were supported by pharmaceutical industry and 22 (46%) by the WHO or the US Centers for Disease Control and Prevention (CDC).

### Cost-of-illness studies

**Fig 2:**
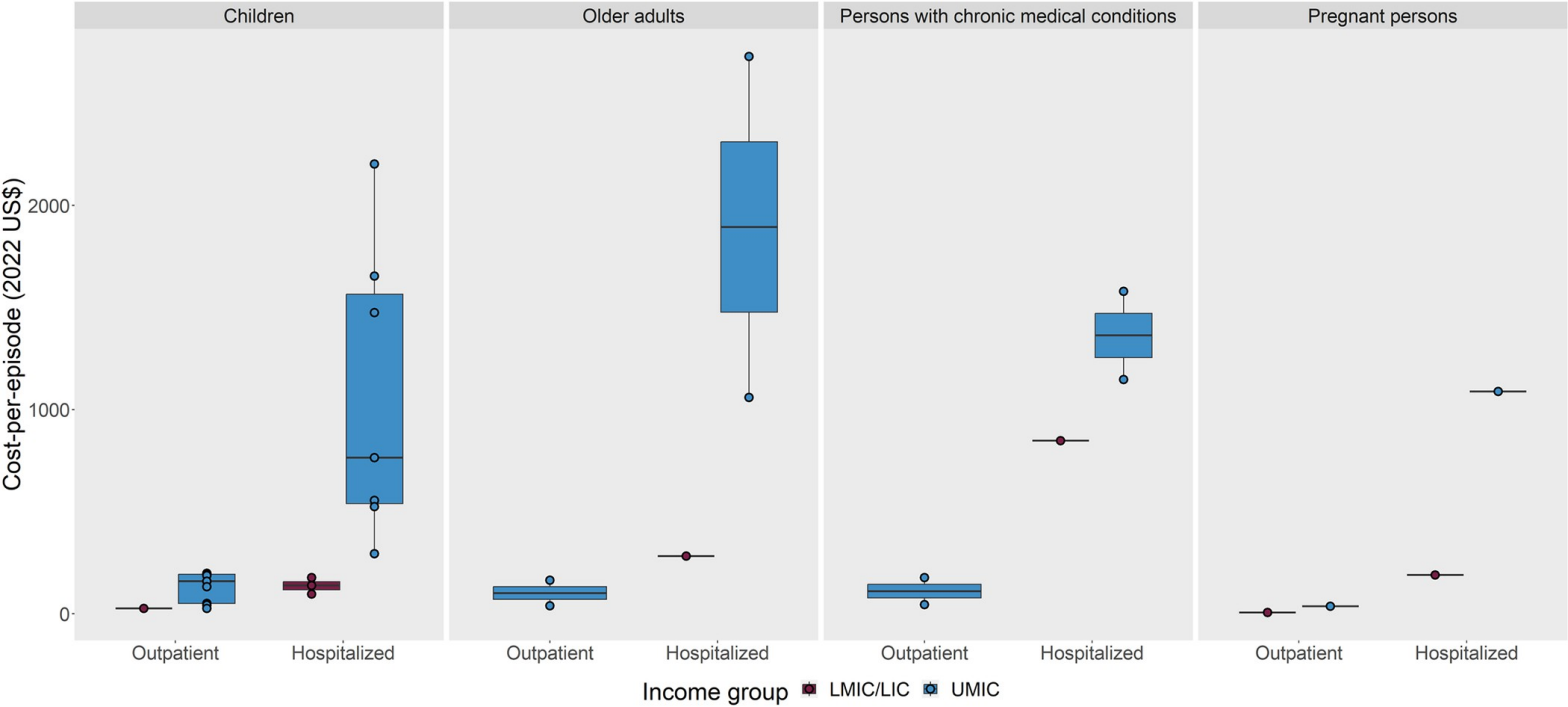
Total costs-per-episode^1^ of influenza illness, by disease severity (outpatient vs. hospitalized)^2^, income group, and Strategic Advisory Committee of Experts on Immunization (SAGE) target group^3^, in low- and middle-income countries. Plot representation: The horizontal line inside the box represents the median. The lower and upper borders of the box represent the 25th and 75th percentiles, respectively. The whiskers indicate 1.5 times the interquartile range from the lower and upper borders of the box. The cost-per-episode^1^ reported in each study is depicted as a filled dot. Costs from low-income and lower-middle income countries are combined as 1 group (“LMIC/LIC”) and shown in magenta; costs from upper-middle income countries are in blue. The group “Children” is inclusive of children aged <18 years; “Older adults” is inclusive of adults aged ≥60 years. All costs are presented in 2022 US$. LIC, low-income country; LMIC, lower-middle income country; UMIC, upper-middle income country; US$, US Dollars. ^1^Total costs inclusive of direct and indirect costs; direct costs were all medical and non-medical costs directly attributable to patient care. Indirect costs were all costs not directly attributable to patient care (e.g., lost earnings or lost productivity). Median costs were preferentially abstracted from source publications; if unavailable, mean costs were abstracted. ^2^No included papers reported hospitalization costs for older adults or persons with chronic medical conditions in LMIC/LIC. ^3^No cost-of-illness papers were identified for healthcare workers or individuals in congregate living settings in low- and middle-income countries.

The cost-per-episode of influenza illness ranged widely across studies (**Fig 2**). Twenty-four studies presented data about cost-per-episode, representing 8 UMICs (China [31–39], Colombia [40,41], Kazakhstan [42], Mexico [43], Panama [44], Romania [42], South Africa [45,46], and Thailand [47]), 6 LMICs (China [based on classification during study period] [48], El Salvador [44], India [49], Kenya [50], Ukraine [42], and Vietnam [51,52]), and 2 LICs (Bangladesh [53] and Mali [54]) (**S4 Table**). Among the general population, the total cost-per-episode for outpatient visits, inclusive of direct and indirect costs, ranged from $6.24 to 155.92 (2022 US$); the total cost-per-episode for hospitalizations ranged from $106.85 to 1,617.14. Among SAGE target groups, total cost-per-episode of outpatient visits and hospitalizations was $25.92 to 198.13 and $95.15 to 2,202.74 for children, $38.17 to 164.52 and $282.37 to 2,729.25 for older adults, $44.13 to 176.79 and $847.60 to 1,578.86 for persons with chronic medical conditions, and $5.45 to 36.97 and $189.98 to 1,088.92 for pregnant persons. Costs across all target groups were generally higher in UMICs than in LMICs/LICs (**Fig 2**). Indirect costs comprised a greater proportion of the total costs of outpatient visits compared with hospitalizations and a greater proportion of total costs in LMICs/LICs compared with UMICs (**S2 Fig**). Details on costs abstracted from each study are described in **S4 Table.**

Four studies evaluated the cost-per-episode for multiple SAGE target groups [33,37,46,51]. Studies from China and Vietnam found higher hospitalization costs among older adults compared with children [33,37,51], as well as higher costs associated with chronic medical conditions across age groups [33,37]. In South Africa, total economic burden after incorporating rates of illness was highest for persons with chronic medical conditions, followed by children, older adults, and pregnant persons [46]. Across all studies, characteristics that impacted cost-of-illness included urbanicity (rural versus urban) [33,37,48], facility type (public versus private or level of care provision) [33,53], and influenza season or circulating virus type [35,38].

Seven of the 23 studies reported a national economic burden of influenza illness for either the general population or specific SAGE target groups (**Table 2**), representing 3 UMICs (China [39], Romania [42], and South Africa [45,46]), 2 LMICs (Kenya [50] and Ukraine [42]), and 1 LIC (Bangladesh [53]). Total annual costs of influenza illness in studies evaluating the general population (no specified SAGE target group) were equivalent to 0.02% to 0.19% of the national GDP and 0.32% to 7.16% of the national health expenditure; costs for any single target group were <0.01% to 0.02% of the national GDP and 0.01% to 0.42% of the national health expenditure. The highest total costs, as a percent of GDP and national health expenditure, were reported from Bangladesh [53]. Three studies accounted for non-medically attended illnesses in the estimation of national economic burden [42,45,46].

**Table 2:** National economic burden of influenza illness, by Strategic Advisory Committee of Experts on Immunization (SAGE) target group^1^, in low- and middle-income countries.

Income group	Study	Country	Target group details	Data source for national extrapolation	Study period	Perspective	Total annual cost (2022 US$, millions)^2^	Total annual cost as % of total national GDP^2^^,^^3^	Total annual cost as % of national health expenditure^2^^,^^4^
**General population**									
UMIC	Tempia, 2019 [45]	South Africa	All ages	7 sentinel hospitals and 2 clinics	2013–15	Societal	$322.62^5^	0.09%	1.16%
UMIC	Gong, 2021 [39]	China	All ages	Not reported	2006–19	Societal	$4,249.40	0.03%	0.55%
LMIC^6^	Emukule, 2019 [50]	Kenya	All ages	4 sentinel hospitals and 1 clinic	2013–14	Societal	$10.76–38.26	0.02%–0.06%	0.32%–1.14%
LIC^7^	Bhuiyan, 2014 [53]	Bangladesh	All ages	4 sentinel hospitals	2010	Societal	$219.68	0.19%	7.16%
**Children** ^8^									
UMIC	Tempia, 2020 [46]	South Africa	6–59 months	7 sentinel hospitals and 2 clinics	2013–15	Societal	$39.97^5^	0.01%	0.14%
LMIC^6^	Emukule, 2019 [50]	Kenya	<5 years	4 sentinel hospitals and 1 clinic	2013–14	Societal	$6.19–14.21	0.01%–0.02%	0.18%–0.42%
**Older adults**									
UMIC	Kovacs, 2014 [42]	Romania	≥65 years	26 sentinel hospitals	2011–12	Payer^9^	$0.68^5^	<0.01%	0.01%
UMIC	Tempia, 2020 [46]	South Africa	≥65 years	7 sentinel hospitals and 2 clinics	2013–15	Societal	$18.75	0.01%	0.07%
LMIC	Kovacs, 2014 [42]	Ukraine	≥65 years	10 sentinel hospitals	2011–12	Payer^9^	$0.79	<0.01%	0.01%
**Persons with chronic medical conditions**									
UMIC	Tempia, 2020 [46]	South Africa	5–64 years with HIV, TB, or other UMC	7 sentinel hospitals and 2 clinics	2013–15	Societal	$102.15^5^	0.03%	0.37%
**Pregnant persons**									
UMIC	Tempia, 2020 [46]	South Africa	NA	7 sentinel hospitals and 2 clinics	2013–15	Societal	$7.24^5^	<0.01%	0.03%

^1^No cost-of-illness papers were identified for healthcare workers or individuals in congregate living settings in low- and middle-income countries.

^2^Calculated values not reported in source publication.

^3^National GDP obtained from World Bank [24], reported for final year of study period.

^4^Current Health Expenditure obtained from World Health Organization [27], reported for final year of study period.

^5^Included estimation of non-medically attended illnesses.

^6^Kenya changed classification from LIC to LMIC in 2014, during the study period [24], and was thus classified as LMIC.

^7^Bangladesh changed classification from LIC to LMIC in 2014, after the study period [24], and was thus classified as LIC.

^8^Although SAGE recommendations specifically reference children aged <5 years [1], publications with data for children aged <18 years were included.

^9^No indirect costs were included in the total estimate because of study perspective. The specific payer was not specified in the source publication.

GDP, gross domestic product; HIV, human immunodeficiency virus; ILI, influenza-like illness; LCI, laboratory-confirmed influenza; LIC, low-income country; LMIC, lower-middle income country; NA, not applicable; SARI, severe acute respiratory infection; TB, tuberculosis; UMC, underlying medical condition; UMIC, upper-middle income country; US$, US Dollars.

### Program cost studies

Five studies evaluated the cost of influenza vaccination programs (**Table 3**): 4 with findings from UMICs (Albania [55], China [56], South Africa [57], and Thailand [58]) and 1 from an LIC (Malawi [59]). Of these, 2 evaluated the cost of a program targeting pregnant persons [58,59], 1 evaluated a program targeting healthcare workers [55], and 2 evaluated programs targeting multiple SAGE target groups (older adults, persons with chronic medical conditions, children aged <5 years, pregnant persons, and healthcare workers in China [56] and older adults, pregnant persons, and persons with chronic medical conditions and human immunodeficiency virus (HIV) in South Africa [57]). Three studies used the WHO SIICT [12,55,57,59]. The total annual cost of program was equivalent to <0.01% to 0.02% of the national GDP and 0.06% to 4.78% of the national health expenditure; the highest proportion of health expenditure was reported in the study vaccinating the greatest number of target groups [56]. Vaccine procurement, when vaccine was purchased by the government, represented a large proportion of total costs in both Albania (89% financial and 44% economic) [55] and South Africa (99% financial and 37% economic) [57]. Additionally, when vaccine was donated, the value of vaccine procurement represented 82% of economic costs in Malawi [59]. Across studies, the total cost per dose administered ranged from $0.62 to 5.20 (financial) and $0.81 to 13.72 (economic), inclusive of vaccine purchase or donation.

**Table 3:** Costs of national influenza vaccination programs in low- and middle-income countries.

Study characteristics							Scenario characteristics			Base scenario results			
Income group	Study	Country	Program year(s) costed^1^	Costing tool/method used	SAGE target group	Perspective	Base scenario: vaccine formulation and cost assumptions	Base scenario: coverage	Additional scenarios modeled	Total annual cost as % of total national GDP^2^^,^^3^	Total annual cost as % of national health expenditure^4^	Vaccine procurement cost^5^ as % of total cost	Cost per dose administered (2022 US$)^3^
UMIC	Yang 2016 [56]	China	1 year (2015)	Budget impact analysis using secondary data inputs	**Multiple**: (1) older adults ≥60y; (2) persons with chronic medical conditions; (3) children <5y; (4) pregnant persons; and (5) healthcare workers	Govt.	TIV: $4.87/dose for 0.25 ml formulation (infants aged 6–35 months) and $7.17/dose for 0.50 ml formulation (all ages >35 months)	20%	Varied vaccine uptake and wastage rate	0.01%	4.78%	NR	NR
UMIC	Fraser, 2022 [57]	South Africa	1 season (2018–19)	SIICT^6^ micro-costing approach	**Multiple**: (1) older adults >65y; (2) pregnant persons; (3) persons with chronic medical conditions and HIV	Govt. (public payer)	TIV: $3.04/dose for government-purchased single-dose vial presentation	4.6% total across target groups	Varied vaccine coverage and product (TIV to QIV)	<0.01%	0.05%	99% (financial), 37% (economic)	$6.45 (economic) excluding vaccine procurement; $3.74 (financial), $10.09 (economic) including vaccine procurement
UMIC	Pallas 2020 [55]	Albania	1 season (2018–19)	SIICT^6^ micro-costing approach	**Healthcare workers**	Govt.	TIV: $4.54/dose for donated single-dose vial presentation; $5.30/dose for government-purchased single-dose pre-filled syringe presentation	70%	Varied vaccine coverage, vaccine presentation, and purchase price	<0.01%	0.06%	89% (financial), 44% (economic)	$0.56 (financial), $7.68 (economic) excluding vaccine procurement; $5.20 (financial), $13.72 (economic) including vaccine procurement
UMIC	Riewpaiboon 2021 [58]	Thailand	1 year (2016)	Micro-costing approach sampling district health facilities	**Pregnant persons**	Health system	Unspecified seasonal vaccine: cost not reported	NR	NR	NR	NR	NR	$0.81–11.70 (economic) range across health facilities
LIC	Pecenka 2017 [59]	Malawi	5 years (2018–22)	SIICT^6^ micro-costing approach	**Pregnant persons**	Govt.	Unspecified seasonal vaccine: $0/dose (financial cost) and $2.90/dose (economic cost) for donated single-dose pre-filled vaccine presentation	47%	Varied vaccine uptake and purchase price	0.02%	1.07%	1% (financial), 82% (economic) assuming donated vaccine	$0.62 (financial), $5.46 (economic) assuming donated vaccine

^1^Program years could be defined by calendar year or influenza season (annual seasonal epidemic period) and are designated accordingly.

^2^National GDP obtained from World Bank [24], reported for final program year costed. Economic program costs were used for the calculation if both financial and economic total costs were reported.

^3^Calculated values not reported in source publication.

^4^Percent of government health expenditure was obtained from the source publication if reported (Pallas and Yang); else, Domestic General Government Health Expenditure obtained from World Health Organization [27]. Economic program costs were used for the calculation if both financial and economic total costs were reported.

^5^Vaccine procurement cost includes cost of vaccine and vaccination supplies.

^6^The “WHO Flutool for planning and costing maternal influenza vaccination” was originally released in 2016; in 2019, the updated “Flutool plus–Seasonal Influenza Immunization Costing Tool (SIICT)” was released and allowed for influenza vaccination cost estimation in additional target groups [12]. Both tools are referred to as “SIICT” in this table.

GDP, gross domestic product; Govt., government; LIC, low-income country; ml, milliliter; NR, not reported; QIV, quadrivalent influenza vaccine; SAGE, Strategic Advisory Group of Experts on Immunization; SIICT, Seasonal Influenza Immunization Costing Tool; TIV, trivalent influenza vaccine; UMIC, upper-middle income country; US$, US Dollars; WHO, World Health Organization; y, years.

### Cost-effectiveness studies

Twenty-six studies presented data on cost-effectiveness of influenza vaccination (**S5 Table**), representing 8 UMICs (Argentina [60], China [61–67], Colombia [68], Malaysia [69], Mexico [70–72], South Africa [73–75], Thailand [76–80], and Turkiye [81]), 4 LMICs (Kenya [82], Lao PDR [83], Ukraine [84], and Vietnam [73]), and 1 LIC (Mali [54]). Twenty-two (85%) studies evaluated influenza vaccine introduction (i.e., vaccination compared with no vaccination), 2 evaluated the effect of increased vaccination coverage on an existing program, and 2 evaluated combinations of new introduction and increased coverage for different target groups. Cost-effectiveness thresholds varied greatly across studies; most (*n* = 16/26; 62%) used a threshold within 1 to 3 times the GDP per capita, 3 (12%) used other country-specific thresholds, 1 (4%) intentionally did not report a threshold (instead, cost-effectiveness acceptability curves were presented over a range of willingness-to-pay thresholds [73]), and the remaining 6 (23%) did not provide any details about thresholds.

**Fig 3:**
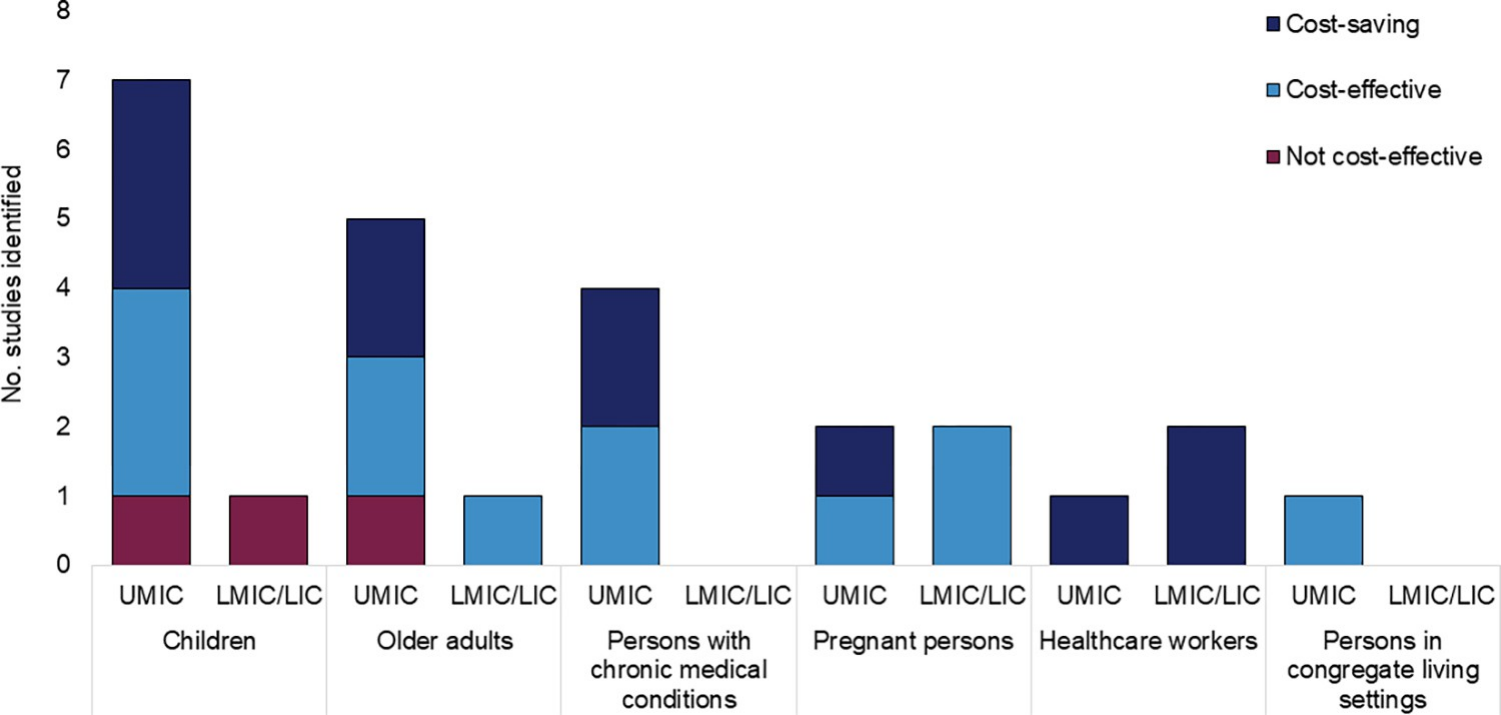
Cost-effectiveness results of studies evaluating influenza vaccination^1^, by Strategic Advisory Committee of Experts on Immunization (SAGE) target group, in low- and middle-income countries. Plot representation: Bars represent the number of studies identified by target group and income group. Results from low-income and lower-middle income countries are combined and analyzed as 1 group, designated “LMIC/LIC.” Dark blue bars depict the number of studies that interpreted a result as “cost-saving,” light blue bars depict the number of studies that interpreted a result as “cost-effective,” and magenta bars depict the number of studies that interpreted a result as not cost-effective. Categorization is based on the interpretation provided in the original study; if any modeled intervention was interpreted as cost-saving (ICER<0), the study was characterized as “cost-saving” and if any modeled intervention was interpreted as cost-effective, the study was characterized as “cost-effective.” Interpretations of highly cost-effective and cost-effective were both combined as “cost-effective.” Details on each modeled scenario are provided in S5 Table. The group “Children” is inclusive of children aged <18 years; “Older adults” is inclusive of adults aged ≥60 years. LIC, low-income country; LMIC, lower-middle income country; UMIC, upper-middle income country. ^1^Only includes studies comparing cost-effectiveness of influenza vaccination vs. no vaccination. Additional studies examining cost-effectiveness of modifications to a current vaccination program (e.g., increased coverage) are described in S5 Table.

Among the 22 studies that evaluated the cost-effectiveness of vaccine introduction, 8 provided results for children, 6 for older adults, 4 for persons with chronic conditions, 4 for pregnant persons, 3 for healthcare workers, and 1 for persons in congregate living settings, summing to 26 target-group-specific scenarios modeled. Most (23/26; 88%) interpreted at least 1 modeled scenario for each SAGE target group as either cost-effective (based on designated cost-effectiveness threshold) or cost-saving (ICER<0) (**Fig 3**). The number of studies that identified results as cost-saving were 3/8 (38%) for children, 2/6 (33%) for older adults, 2/4 (50%) for persons with chronic medical conditions, 1/4 (25%) for pregnant persons, and 3/3 (100%) for healthcare workers. Similarly, the number of studies that identified results as cost-effective were 3/8 (38%) for children, 3/6 (50%) for older adults, 2/4 (50%) for persons with chronic medical conditions, 3/4 (75%) for pregnant persons, and 1/1 (100%) for persons in congregate living settings. Only 3 studies interpreted all modeled scenarios for a particular target group as not cost-effective: 2/8 (25%) evaluating cost-effectiveness among children [75,82] and 1/6 (17%) among older adults [66].

Two studies assessed the cost-effectiveness of influenza vaccine introduction for multiple target groups; of these, the target groups with the greatest value-for-money were healthcare workers in Laos (cost-saving; other groups evaluated were pregnant persons and older adults, found to be cost-effective) [83], and pregnant persons and persons with chronic medical conditions in South Africa (cost saving; other groups evaluated were older adults, found to be cost-effective, and children, not found to be cost-effective) [75]. Across all studies, the 3 variables most commonly identified to influence the ICER in sensitivity analyses were annual incidence of influenza (*n* = 10 studies) [54,60,61,67,73–75,78,81,83], vaccine effectiveness (*n* = 10 studies) [60,65,67,68,74–77,81,83], and cost of vaccine (*n* = 6 studies) [54,74,75,77,78,82]; some studies demonstrated that variation in the attack rate [61,73,75,78] or vaccine effectiveness [75] could change the interpretation of cost-effectiveness results (cost-saving, cost-effective, or not-cost-effective).

Additionally, 7 papers looked at different prioritization strategies within SAGE target groups by underlying health conditions [74,76] or age [60,61,77,79,82]; among pregnant persons in South Africa, prioritization of people living with HIV reduced the ICER (though not statistically significant) [74], and among persons with underlying coronary heart disease in Thailand, restricting to only persons with angina reduced the ICER, whereas restricting to persons with cardiac arrest/myocardial infarction increased the ICER (no longer cost-effective) [76]. Results by age were also mixed; 2 studies among children found lower ICERs for vaccinating younger children (6 to 23 months versus 2 to 5 years or 6 to 14 years in Kenya [82] and 6 to 59 months versus 5 to 14 years in China [61]), 2 studies among children found lower ICERs for vaccinating wider age ranges (6 months to 5 years versus 6 to 23 months or 6 to 36 months in Argentina [60]) or older children (12 to 17 years versus 2 to 5 years or 6 to 11 years in Thailand [77]), and a study among persons with underlying heart disease in Thailand found a lower ICER for persons aged ≥50 years compared with ≥40 years or ≥60 years [79].

## Discussion

The 54 studies identified in this review suggest an increased momentum to generate economic evidence about influenza illness and vaccination from low- and middle-income countries during 2012 to 2022; a previous review using a similar search strategy identified only 22 cost-of-illness or cost-effectiveness studies from low- and middle-income countries published as of 2012 [15], and another identified 9 cost-effectiveness/cost-benefit/cost-utility studies published as of 2011 [13]. The release of updated tools and guidance by the WHO, as well as technical and financial support by the WHO, CDC, and other international partners, has facilitated this expansion of the evidence base, emphasizing the utility of global and multinational collaborations in strengthening influenza vaccination programs worldwide. Studies included in this review were generally of good quality based on their quality assessment scores; however, we identified substantial variability in methodologies and approaches. Although methods for meta-analysis of economic data are available [85] and have been used in other reviews that focus predominantly on high-income settings [17], we did not conduct these analyses because of study heterogeneity.

Recent additions to the literature since 2012 include studies from LMIC/LICs, studies representing sub-Saharan Africa, South Asia, and middle-income European countries, and studies focused on pregnant persons; none of these were represented in the previous reviews, which only identified data from UMICs in East Asia, Latin America, and Europe [13,15]. However, there are still disparities in available data by income group and region; LICs are still very underrepresented, and no studies from low- and middle-income countries in the Middle East and North Africa region were identified in our review. As of a global survey in 2018, very few LICs (2/31; 6%) reported having a national influenza vaccine policy in place [2]; absence of a vaccination program could in part explain their underrepresentation in the literature, but also underscores the importance of generating policy-relevant data on cost-of-illness and projected costs and cost-effectiveness of vaccination. By comparison, 78% (45/58) of UMICs and 39% (18/46) LMICS had an influenza vaccine policy in 2018 [2].

Additionally, pregnant persons, healthcare workers, and persons in congregate living settings remain especially underrepresented in economic evaluations. Healthcare workers are of particular interest because of the potential benefit of vaccination to themselves and the greater health system [86]. To date, global literature about cost-effectiveness and other evidence for influenza vaccination among healthcare workers remains limited [86,87], but notably, we identified cost-saving results for healthcare worker vaccination in Lao PDR [83], Malaysia [69], and Ukraine [84], suggesting high value-for-money. Additional data are needed to strengthen the evidence to optimize influenza vaccination in this target group.

Among all cost-of-illness studies, we found that the cost-per-episode estimates for influenza outpatient visits and hospitalizations varied widely. Per-episode costs were generally greater in higher income settings (i.e., UMICs compared with LMICs/LICs), likely reflecting higher costs of care, but the national economic burden among the general population, which ranged from 0.01% to 7% of the national health expenditure, was highest in an LIC (Bangladesh; 7% [53]). For comparison, a prior review indicated that the economic burden as a percentage of national GDP, in high-income countries and UMICs in North America, Europe, Asia, and Australia, ranged from 0.01% to 0.14% [15]. More studies from LICs are needed to further evaluate disparities among income groups.

Many studies used data from ILI/SARI sentinel surveillance sites for estimating economic burden; these surveillance systems can serve as a valuable data source but are not typically designed to capture non-medically attended illnesses [88] or non-respiratory disease outcomes [89], thereby underestimating the true economic burden of influenza. In South Africa for example, estimates obtained among patients meeting a SARI/ILI case definition underestimated the total economic burden by approximately 65% [45]; thus, comprehensive strategies and innovative strategies are needed to better characterize economic burden. Finally, characteristics of the underlying population in a particular setting, such as age structure and prevalence of underlying medical conditions, might affect costs across target groups; for example, in South Africa, where the highest burden was in individuals with chronic medical conditions, this was impacted by HIV and tuberculosis prevalence in the population [45,46]. Thus, economic evaluations that address multiple target groups in a particular setting, rather than a single target group, can provide valuable evidence to inform local vaccination policy; given limited resources for vaccination programs, such comparisons could assist with target group prioritization.

We identified only 5 program cost studies, indicating a need for more evaluations in low- and middle-income countries. Vaccine delivery cost studies can provide direct evidence to policymakers to make decisions on vaccine introduction, plan budgets and financing strategies for rollout, and identify efficiencies in service delivery [90]. In fact, the WHO SIICT [12] and other costing methods can be used even in the absence of an existing program, as performed in Malawi [59]. The 5 studies that we identified indicated that influenza vaccination programs generally cost a small fraction compared to the national GDP (≤0.02% in these studies) or national health expenditure (≤1% per each individual target group covered in these studies) [55–59]. Vaccine procurement was a major driver of program costs in all 3 studies that disaggregated this component, representing 82% of economic costs (including the value of donated resources) of the hypothetical maternal vaccination program using donated vaccine in Malawi [59], 99% of financial and 37% of economic costs of a vaccination program utilizing government-procured vaccines for multiple target groups in South Africa [57], and 89% of financial and 44% of economic costs of a healthcare worker vaccination program utilizing a combination of government-procured and donated vaccines in Albania [55]. This underscores the importance of sustainable financing and procurement strategies to support access to influenza vaccines and enable successful program implementation, consistent with lessons learned from other vaccine introductions [91]. In the 2 studies that presented costs exclusive of vaccine procurement ($7.68 economic cost-per-dose for healthcare workers in Albania [55] and $6.45 economic cost-per-dose for multiple target groups in South Africa [57]), the costs for influenza vaccination were generally higher than the estimated incremental cost to deliver a single, newly introduced vaccine (e.g., pneumococcal conjugate vaccine or rotavirus vaccine) in low-income countries ($0.57 to 1.63 in 2022 US$) [92], likely because the costs for delivering vaccine to influenza target groups rely on different systems and infrastructure than routine immunization delivery for children. Again, as costs may vary across target groups, evaluating program costs in multiple groups within a given country context could provide useful data for resource prioritization.

Among cost-effectiveness studies identified in this review, most reported at least 1 cost-saving or cost-effective vaccination scenario per target group assessed; however, results were significantly impacted by variables such as influenza incidence, vaccine effectiveness, cost of vaccine, and vaccine coverage, as well as by prioritization within target groups (e.g., by age or specific underlying health conditions). Strategies to address this variability include use of at least 5 years of data to assess disease burden, if available, and use of sensitivity analyses among ranges of plausible values, for example, including vaccine effectiveness estimates from years with high and low antigenic match of vaccine with circulating viruses [11]. Future studies could use innovative approaches to more completely characterize the total disease and economic burden of influenza, as well as additional endpoints for vaccine effectiveness (illness attenuation) and indirect protection from vaccination [7]. Finally, the use of appropriate cost-effectiveness thresholds in low- and middle-income settings warrants further discussion [93,94]. In our review, among only 3 studies that did not identify any cost-effective scenarios, 2 used a cost-effectiveness threshold less than GDP per capita [75,82]; however, using the GDP per capita would have resulted in a cost-effective result in both. Use of context-specific thresholds reflecting local preferences [95], such as local health opportunity costs [96], might provide more valuable information to guide investment decisions than thresholds of 1 to 3 times GDP per capita [93,94,97,98]. As indicated in the WHO guidance for economic evaluations for immunization programs, if willingness-to-pay values are not available for a given country, cost-effectiveness results should be shown for a range of willingness-to-pay values, along with the vaccine price on which they are based [99].

This review is subject to several notable limitations. First, the inclusion/exclusion criteria used (e.g., estimates derived from LCI or ILI/SARI case definition; no comparison of vaccine formulations) undercount the total number of economic studies from low- and middle-income countries within the past 10 years. Multiple other studies have evaluated costs of acute respiratory illness, of which influenza is an important etiology, or addressed other economic questions, such as the cost-effectiveness of quadrivalent versus trivalent vaccine [23,100], and were not captured here. Influenza illness might also present as non-respiratory outcomes [89], and thus the economic burden of influenza is underestimated in most studies that restrict to syndromic surveillance for ILI/SARI [88]. Second, although we conducted the search using multiple databases and considered publications in any language eligible for inclusion, we might have missed studies published in national or regional journals. Third, target group definitions vary across countries, with variation in age cut-offs for children and older adults and prioritization of specific chronic medical conditions, but all results per target group were summarized together in this review because of the small numbers of publications, potentially missing nuances of within-group differences. Relatedly, although SAGE recommendations specifically reference children aged <5 years [1], all publications with data for children and adolescents aged <18 years were included. Finally, we found substantial heterogeneity in the methodology and data inputs used across studies; in addition, influenza itself intrinsically varies in annual incidence, disease severity, and vaccine effectiveness across seasons. As previously discussed, we did not conduct meta-analyses because of this variability, though methods for meta-analysis of economic data are available [85] and have been used in other reviews that focus predominantly on high-income settings [21].

This review also uncovered opportunities to provide evidence about policy-relevant questions that currently have limited evidence. First, we only identified 1 study taking an employer payer’s perspective [69]; additional studies utilizing this approach could provide valuable policy-relevant information to encourage vaccination among employees or to encourage employer-supported vaccination programs [101] as a pathway to broader influenza vaccine availability. Second, we only identified 1 included study that evaluated cost-effectiveness of influenza vaccination coadministered with another vaccine (pneumococcal vaccine) [68]; a few additional studies addressing coadministration were excluded because they did not provide results for influenza vaccination alone. Given opportunities to coadminister influenza vaccine with other vaccines across the life course, including COVID-19 vaccine [102], evaluation of shared costs in program cost or cost-effectiveness studies might incentivize integrated vaccine implementation. Third, we found only 2 studies that considered non-respiratory disease outcomes (cardiovascular disease events) in cost-effectiveness analyses, both among persons with underlying heart disease in Thailand [76,79]; as previously discussed, inclusion of non-respiratory disease outcomes could better characterize the full impact of influenza vaccination [89]. Finally, innovative strategies might address the broader impact of vaccines, such as impact on childhood development, household behavior, economic growth, political stability, and health equity [103,104].

Continued evaluation of costs and cost-effectiveness is useful to drive evidence-based vaccine policy development, implementation, refinement, and global investment in influenza vaccination. In this review, we documented an increased number of economic evaluations on influenza illness and vaccination from low- and middle-income countries during 2012 to 2022 compared with prior years. Additional studies from low-income countries and underrepresented target groups (e.g., pregnant persons, healthcare workers, and persons in congregate living settings) would strengthen the evidence regarding value-for-money. Standardization of research agenda [1] and methodology across future evaluations, including considerations to capture the full spectrum of influenza-associated illness, could allow for pooled estimates and meta-analyses. Global, regional, and country-specific data on the economics of vaccination, including costs of vaccination programs, costs of avertable illnesses, and cost-effectiveness, are instrumental for policymaking and resource allocation for expanded and sustainable influenza vaccination programs.

## Supporting information

S1 PRISMA Checklist PRISMA 2020 Main Checklist. (DOCX)

S1 Fig Distribution of modified Consolidated Health Economic Evaluation Reporting Standard (CHEERS) quality assessment scores. Plot representation: The horizontal line inside the box represents the median. The lower and upper borders of the box represent the 25th and 75th percentiles, respectively. The whiskers indicate 1.5 times the interquartile range from the lower and upper borders of the box. All CHEERS scores are presented as a percent of total possible score; the full CHEERS criteria assessment [28] was performed for cost-effectiveness studies and a modified set of relevant criteria were assessed for cost-of-illness and cost-of-program studies, as explained in S2 Table. CHEERS, Consolidated Health Economic Evaluation Reporting Standard.
(TIFF)

S2 Fig Contribution of direct and indirect costs to costs of influenza outpatient visits and hospitalizations, by target group, in low- and middle-income countries. Plot representation: Vertical bars represent the contribution (as percent of total) of direct costs (blue) and indirect costs (gray) to total outpatient visit costs (left column) and hospitalization costs (right column), by target group. Direct costs were all medical and non-medical costs directly attributable to patient care, as reported in the study. Indirect costs were all costs not directly attributable to patient care (e.g., lost earnings or lost productivity). The group “Children” is inclusive of children aged <18 years; “Older adults” is inclusive of adults aged ≥60 years. LIC, low-income country; LMIC, lower-middle income country; UMIC, upper-middle income country.
(TIF)

S1 Table Search terms for systematic review, by database. (DOCX)

S2 Table Modified Consolidated Health Economic Evaluation Reporting Standard (CHEERS) criteria^1^ used for quality assessment. DALY, disability-adjusted life years; QALY, quality-adjusted life years. ^**1**^The full CHEERS criteria assessment [28] was performed for cost-effectiveness studies; a modified set of relevant criteria were assessed for cost-of-illness and cost-of-program studies.
(DOCX)

S3 Table Description of included studies, by country. LIC, low-income country; LMIC, lower-middle income country; SAGE, Strategic Advisory Committee of Experts on Immunization; UMIC, upper-middle income country; US$, US Dollars. ^1^Although SAGE recommendations specifically reference children aged <5 years [1], publications with data for children aged <18 years were included. ^2^Publications with data for adults aged ≥60 years. ^3^Bangladesh changed classification from LIC to LMIC in 2014 [24], after the study period [53], and was thus classified as LIC. ^4^China was classified as both UMIC and LMIC corresponding to studies before and after an upward change in World Bank classification in 2010. ^5^Kenya changed classification from LIC to LMIC in 2014 [24], during the study period for 1 study [50], and was thus classified as LMIC for both studies.
(DOCX)

S4 Table Costs of influenza illness^1^, by Strategic Advisory Committee of Experts on Immunization (SAGE) target group^2^ and disease severity (outpatient vs. hospitalized), in low- and middle-income countries. HIV, human immunodeficiency virus; ILI, influenza-like illness; LCI, laboratory-confirmed influenza; LIC, low-income country; LMIC, lower-middle income country; NA, not applicable; NR, not reported; P&I, pneumonia and influenza hospitalization; SARI, severe acute respiratory infection; TB, tuberculosis; UMC, underlying medical condition; UMIC, upper-middle income country; US$, US Dollars. ^1^Median costs were preferentially abstracted from source publications; if unavailable, mean costs were abstracted. ^2^No cost-of-illness papers were identified for healthcare workers or individuals in congregate living settings in low- and middle-income countries. ^3^For source publications presenting results for both LCI and syndromic illness, the results for LCI were used. ^4^Direct costs were all medical and non-medical costs directly attributable to patient care. ^5^Calculated or converted value; not presented in source publication. ^6^Indirect costs were all costs not directly attributable to patient care (e.g., lost earnings or lost productivity). ^7^Direct medical and non-medical costs were summarized separately in the source publication; medians were summed to obtain a total median direct cost. ^8^China changed classification from LMIC to UMIC in 2010 [24], after the study period, and was thus classified as LMIC for this study. ^9^No indirect costs were included in the total estimate because of study perspective. The specific payer was not specified in the source publication. ^10^Kenya changed classification from LIC to LMIC in 2014 [24], during the study period, and was thus classified as LMIC. ^11^Bangladesh changed classification from LIC to LMIC in 2014 [24], after the study period, and was thus classified as LIC. ^12^Included only direct medical costs (no non-medical costs). ^13^Although SAGE recommendations specifically reference children aged <5 years [1], publications with data for children aged <18 years were included. ^14^The full publication study period was 2005–2011; however, 2009–2010 and 2010–2011 were excluded because of H1N1 pandemic activity. Abstracted values represent the median of 2005–2009 annual values. ^15^Cost data were only available for 1 hospitalized LCI case; thus, ILI hospitalization costs were abstracted.
(DOCX)

S5 Table Cost-effectiveness of influenza vaccination^1^, by Strategic Advisory Committee of Experts on Immunization (SAGE) target group, in low- and middle-income countries. DALY, disability-adjusted life year; ICER, incremental cost-effectiveness ratio; Govt, government; HIV, human immunodeficiency virus; LAIV, live attenuated influenza vaccine; LIC, low-income country; LMIC, lower-middle income country; NA, not applicable; NR, not reported; QALY, quality-adjusted life year; QIV, quadrivalent influenza vaccine; TIV, trivalent influenza vaccine; UMIC, upper-middle income country; US$, US Dollars; WTP, willingness-to-pay. ^1^Cost-effectiveness, cost-utility, and cost-benefit analyses were eligible for inclusion if they included a comparison of influenza vaccination vs. either no vaccination or modifications to current vaccination program. Studies that only compared the cost-effectiveness of different influenza vaccine products were not included. ^2^Data for each base-scenario intervention or each perspective assessed are presented in individual rows. Sensitivity analyses are not presented. Vaccine coverage was rounded to the nearest integer. ^3^Calculated or converted value; not presented in source publication. Ranges represent annual seasonal estimates or varying illness attack rate. ^4^Interpretation per source publication. Interpretations of highly cost-effective and cost-effective were both combined as “cost-effective.” ^5^The study authors intentionally did not specify a cost-effectiveness threshold or interpretation because a country-specific threshold was not available. Instead, cost-effectiveness acceptability curves were presented over a range of willingness-to-pay thresholds. ^6^Similar data sources and analysis methods used in both publications; these were counted collectively as one study for Fig 3. ^7^Interpretation of net costs of vaccination (including illness averted) vs. no vaccination. ^8^Although SAGE recommendations specifically reference children aged <5 years [1], publications with data for children aged <18 years were included. ^9^This study also modeled alternative strategies to increase vaccination rates. ^10^This study used a cost-effectiveness threshold for South Africa that reflects the health opportunity cost of health spending. ^11^The age of the hypothetical cohort was based on the mean age of the target population in China (69 years). ^12^Age groups of ≥50 years and ≥60 years were also modeled; only results for ≥40 years are shown as this was inclusive of all other groups. All scenarios were cost-effective. ^13^Medical conditions included diabetes, high blood pressure, morbid obesity, chronic renal failure, asthma, and pregnancy. ^14^Included patients with angina and cardiac arrest/myocardial infarction. ^15^A country-specific threshold of 100,000 Thai Baht was used (rationale not reported). ^16^The 90% uncertainty intervals for the ICER overlapped the cost-effectiveness threshold. ^17^Additional scenarios adjusted for poor access to care and increased severity of disease; all scenarios were cost-effective. ^18^Results were interpreted as cost-effective when the cost per pregnant woman vaccinated was $1.00 or less. ^19^Additional scenarios modeled higher coverage of 30% and 100%; all scenarios were cost-effective.
(DOCX)

## Abbreviations

CHEERS: Consolidated Health Economic Evaluation Reporting Standard
COVID-19: Coronavirus Disease 2019
GDP: gross domestic product
HIV: human immunodeficiency virus
ICER: incremental cost-effectiveness ratio
ILI: influenza-like-illness
IQR: interquartile range
LCI: laboratory-confirmed influenza
LIC: low-income country
LMIC: lower-middle income country
SAGE: Strategic Advisory Group of Experts on Immunization
SARI: severe acute respiratory infection
SIICT: Seasonal Influenza Immunization Costing Tool
UMIC: upper-middle income country
WHO: World Health Organization
