# Supplementary figures and images for: Costs and cost-effectiveness of influenza illness and vaccination in low- and middle-income countries: A systematic review from 2012 to 2022
Source: PLoS Med. 2024 Jan 5;21(1):e1004333. doi: 10.1371/journal.pmed.1004333 (PMC10802964; doi:10.1371/journal.pmed.1004333)

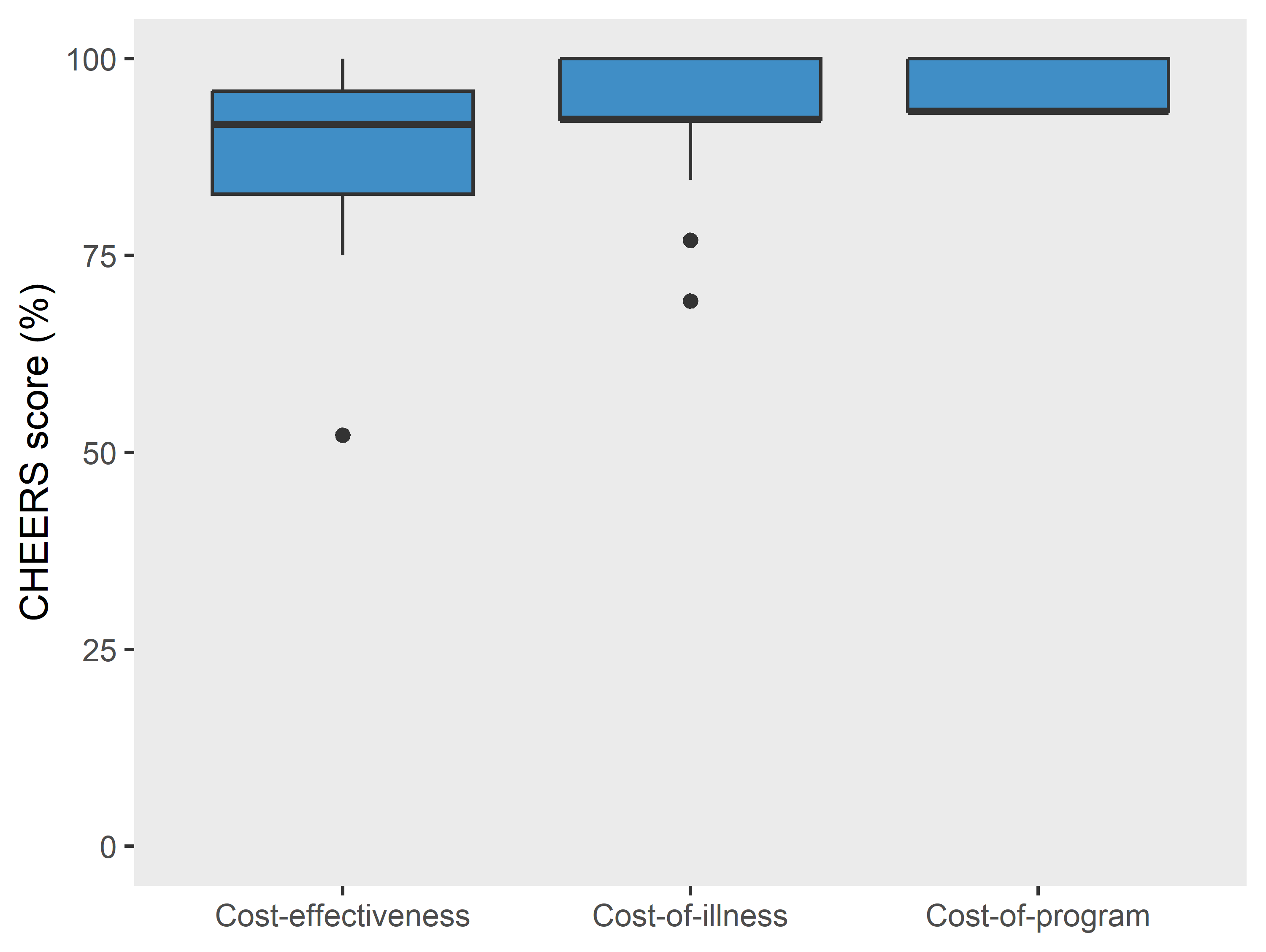

Supplement: S1 Fig — Plot representation: The horizontal line inside the box represents the median. The lower and upper borders of the box represent the 25th and 75th percentiles, respectively. The whiskers indicate 1.5 times the interquartile range from the lower and upper borders of the box. All CHEERS scores are presented as a percent of total possible score; the full CHEERS criteria assessment [28] was performed for cost-effectiveness studies and a modified set of relevant criteria were assessed for cost-of-illness and cost-of-program studies, as explained in S2 Table. CHEERS, Consolidated Health Economic Evaluation Reporting Standard. (TIFF) [file pmed.1004333.s002.tiff]

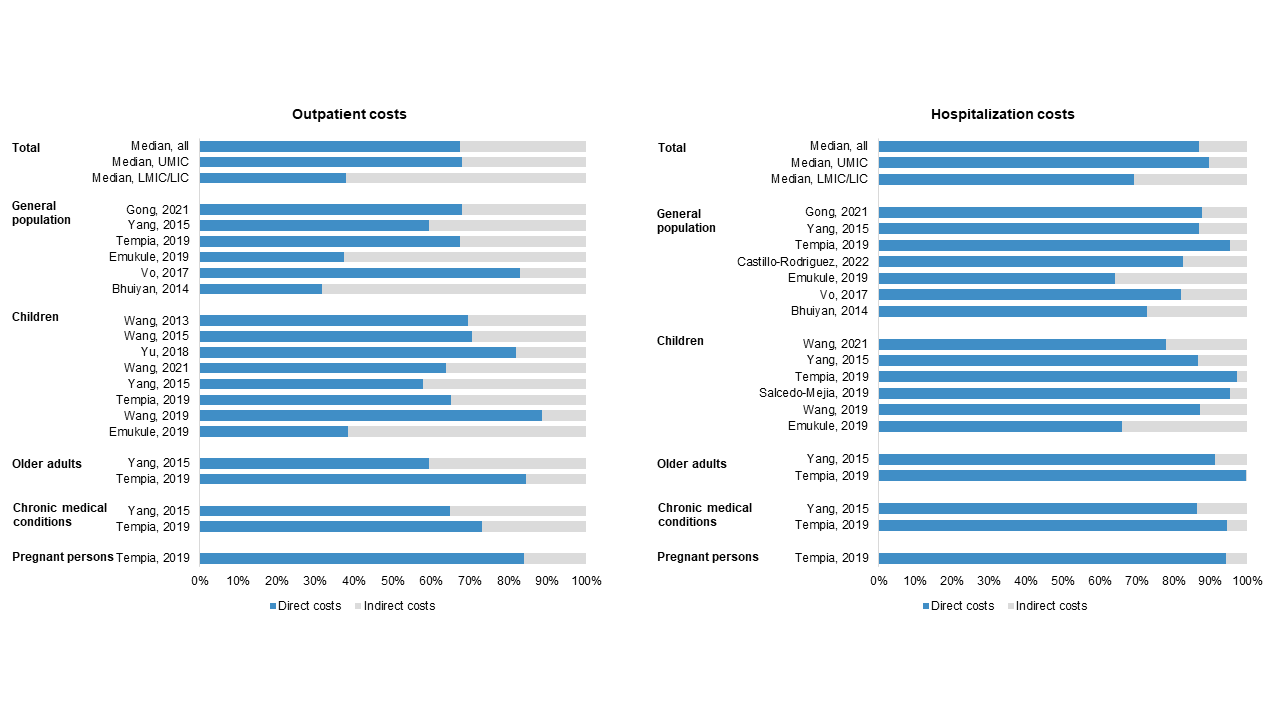

Supplement: S2 Fig — Plot representation: Vertical bars represent the contribution (as percent of total) of direct costs (blue) and indirect costs (gray) to total outpatient visit costs (left column) and hospitalization costs (right column), by target group. Direct costs were all medical and non-medical costs directly attributable to patient care, as reported in the study. Indirect costs were all costs not directly attributable to patient care (e.g., lost earnings or lost productivity). The group “Children” is inclusive of children aged <18 years; “Older adults” is inclusive of adults aged ≥60 years. LIC, low-income country; LMIC, lower-middle income country; UMIC, upper-middle income country. (TIF) [file pmed.1004333.s003.tif]
