# Supplementary material for: Costs and cost-effectiveness of influenza illness and vaccination in low- and middle-income countries: A systematic review from 2012 to 2022
Source: PLoS Med. 2024 Jan 5;21(1):e1004333. doi: 10.1371/journal.pmed.1004333 (PMC10802964; doi:10.1371/journal.pmed.1004333)
Supplement: S1 Table — (DOCX) [file pmed.1004333.s004.docx]

**S1 Table: Search terms for systematic review, by database**

| Database | Strategy | Run Date |
| --- | --- | --- |
| Medline  (OVID)  1946- | (influenza* OR flu OR H1N1)  AND  Cost* OR economic* OR ec.fs  AND  Exp Animals/ NOT exp humans/  2012 – 2021; py=2022 | 01/31/2022 and 10/31/2023 *(for 2022 only)* |
| Embase  (OVID)  1974- | Exp Influenza/ OR (influenza* OR flu OR H1N1).ti,ab.  AND  Cost* OR economic*  AND  Exp Animal/ NOT exp human/  NOT  Conference abstract.pt  Exclude Medline records  2012 – 2021; py=2022 | 01/31/2022 and 10/31/2023 *(for 2022 only)* |
| Cochrane Library | (influenza* OR flu OR H1N1):ti,ab  AND  (Cost* OR economic*):ti,ab  2012 – 2021; py=2022 | 01/31/2022 and 10/31/2023 *(for 2022 only)* |
| CINAHL  (EbscoHost) | (TI (influenza* OR flu OR H1N1)) OR (AB (influenza* OR flu OR H1N1))  AND  (TI (Cost* OR economic*)) OR (AB (Cost* OR economic*))  2012 – 2021; exclude Medline records; py=2022 | 01/31/2022 and 10/31/2023 *(for 2022 only)* |
| Scopus | TITLE-ABS-KEY(influenza* OR flu OR H1N1) AND TITLE-ABS-KEY(Cost* OR economic*) AND NOT INDEX(medline)  2012 – 2021; py=2022 | 01/31/2022 and 10/31/2023 *(for 2022 only)* |
