# Supplementary material for: Costs and cost-effectiveness of influenza illness and vaccination in low- and middle-income countries: A systematic review from 2012 to 2022
Source: PLoS Med. 2024 Jan 5;21(1):e1004333. doi: 10.1371/journal.pmed.1004333 (PMC10802964; doi:10.1371/journal.pmed.1004333)
Supplement: S3 Table — LIC, low-income country; LMIC, lower-middle income country; SAGE, Strategic Advisory Committee of Experts on Immunization; UMIC, upper-middle income country; US$, US Dollars. 1Although SAGE recommendations specifically reference children aged <5 years [1], publications with data for children aged <18 years were included. 2Publications with data for adults aged ≥60 years. 3Bangladesh changed classification from LIC to LMIC in 2014 [24], after the study period [53], and was thus classified as LIC. 4China was classified as both UMIC and LMIC corresponding to studies before and after an upward change in World Bank classification in 2010. 5Kenya changed classification from LIC to LMIC in 2014 [24], during the study period for 1 study [50], and was thus classified as LMIC for both studies. (DOCX) [file pmed.1004333.s006.docx]

**S3 Table: Description of included studies, by country**

| Country | Author, year | Study type | SAGE target group(s) | | | | | | | CHEERS^1^ score | Reference |
| --- | --- | --- | --- | --- | --- | --- | --- | --- | --- | --- | --- |
|  |  |  | None (general population) | Children^2^ | Older adults^3^ | Persons with chronic medical conditions | Pregnant persons | Healthcare workers | Persons in congregate living settings |  |  |
| Albania (UMIC) | Pallas, 2020 | Cost-of-program |  |  |  |  |  | ✓ |  | 100% | [1] |
| Argentina (UMIC) | Giglio, 2012 | Cost-effectiveness (or cost-benefit, cost-utility) |  | ✓ |  |  |  |  |  | 75% | [2] |
| Bangladesh (LIC)^4^ | Bhuiyan, 2014 | Cost-of-illness | ✓ |  |  |  |  |  |  | 92% | [3] |
| China (LMIC, UMIC)^5^ | Chen, 2019 | Cost-effectiveness (or cost-benefit, cost-utility) |  |  | ✓ |  |  |  |  | 92% | [4] |
|  | Gong, 2021 | Cost-of-illness | ✓ |  |  |  |  |  |  | 77% | [5] |
|  | Guo, 2012 | Cost-of-illness | ✓ |  |  |  |  |  |  | 85% | [6] |
|  | Jiang, 2020 | Cost-effectiveness (or cost-benefit, cost-utility) |  |  | ✓ |  |  |  |  | 100% | [7] |
|  | Lai, 2021 | Cost-of-illness |  | ✓ | ✓ | ✓ |  |  |  | 92% | [8] |
|  | Wang, 2013 | Cost-of-illness |  | ✓ |  |  |  |  |  | 85% | [9] |
|  | Wang, 2015 | Cost-of-illness |  | ✓ |  |  |  |  |  | 92% | [10] |
|  | Wang, 2019 | Cost-of-illness |  | ✓ |  |  |  |  |  | 77% | [11] |
|  | Wang, 2021 | Cost-of-illness |  | ✓ |  |  |  |  |  | 100% | [12] |
|  | Wu, 2022 | Cost-effectiveness (or cost-benefit, cost-utility) |  |  | ✓ |  |  |  |  | 96% | [13] |
|  | Yan, 2021 | Cost-effectiveness (or cost-benefit, cost-utility) |  |  | ✓ |  |  |  |  | 92% | [14] |
|  | Yang, 2015 | Cost-of-illness | ✓ | ✓ | ✓ | ✓ |  |  |  | 100% | [15] |
|  | Yang, 2016 | Cost-of-program |  | ✓ | ✓ | ✓ | ✓ | ✓ |  | 93% | [16] |
|  | Yang, 2019 | Cost-effectiveness (or cost-benefit, cost-utility) |  |  |  | ✓ |  |  |  | 96% | [17] |
|  | Yang, 2020 | Cost-effectiveness (or cost-benefit, cost-utility) |  |  | ✓ |  |  |  |  | 100% | [18] |
|  | Yu, 2018 | Cost-of-illness |  | ✓ |  |  |  |  |  | 100% | [19] |
|  | Zhang, 2017 | Cost-of-illness |  | ✓ |  |  |  |  |  | 69% | [20] |
|  | Zhou, 2014 | Cost-effectiveness (or cost-benefit, cost-utility) |  | ✓ |  |  |  |  |  | 100% | [21] |
| Colombia (UMIC) | Castillo-Rodriguez, 2022 | Cost-of-illness | ✓ |  |  |  |  |  |  | 100% | [22] |
|  | Lara, 2018 | Cost-effectiveness (or cost-benefit, cost-utility) |  | ✓ |  |  |  |  |  | 92% | [23] |
|  | Salcedo-Mejia, 2019 | Cost-of-illness |  | ✓ |  |  |  |  |  | 92% | [24] |
| El Salvador (LMIC) | Jara, 2019 | Cost-of-illness |  | ✓ |  |  |  |  |  | 100% | [25] |
| India (LMIC) | Koul, 2019 | Cost-of-illness |  |  |  | ✓ |  |  |  | 92% | [26] |
| Kazakhstan (UMIC) | Kovacs, 2014 | Cost-of-illness |  |  | ✓ |  |  |  |  | 77% | [27] |
| Kenya (LMIC)^6^ | Dawa, 2020 | Cost-effectiveness (or cost-benefit, cost-utility) |  | ✓ |  |  |  |  |  | 96% | [28] |
|  | Emukule, 2019 | Cost-of-illness | ✓ | ✓ |  |  |  |  |  | 100% | [29] |
| Lao PDR (LMIC) | Ortega-Sanchez, 2021 | Cost-effectiveness (or cost-benefit, cost-utility) |  |  | ✓ |  | ✓ | ✓ |  | 100% | [30] |
| Malawi (LIC) | Pecenka, 2017 | Cost-of-program |  |  |  |  | ✓ |  |  | 93% | [31] |
| Malaysia (UMIC) | Tohiar, 2022 | Cost-effectiveness (or cost-benefit, cost-utility) |  |  |  |  |  | ✓ |  | 52% | [32] |
| Mali (LIC) | Orenstein, 2017 | Cost-of-illness **AND** Cost-effectiveness (or cost-benefit, cost-utility) |  |  |  |  | ✓ |  |  | 83% | [33] |
| Mexico (UMIC) | Betancourt-Craviato, 2021 | Cost-effectiveness (or cost-benefit, cost-utility) | ✓ |  |  |  |  |  |  | 78% | [34] |
|  | Falcon-Lezama, 2020 | Cost-effectiveness (or cost-benefit, cost-utility) |  | ✓ |  |  |  |  |  | 87% | [35] |
|  | Reyes-Lopez, 2022 | Cost-of-illness |  | ✓ |  |  |  |  |  | 77% | [36] |
|  | Tapia-Conyer, 2021 | Cost-effectiveness (or cost-benefit, cost-utility) | ✓ | ✓ |  | ✓ |  |  |  | 83% | [37] |
| Panama (UMIC) | Jara, 2019 | Cost-of-illness |  | ✓ |  |  |  |  |  | 100% | [25] |
| Romania (UMIC) | Kovacs, 2014 | Cost-of-illness |  |  | ✓ |  |  |  |  | 77% | [27] |
| South Africa (UMIC) | Biggerstaff, 2019 | Cost-effectiveness (or cost-benefit, cost-utility) |  |  |  |  | ✓ |  |  | 100% | [38] |
|  | Edoka, 2021 | Cost-effectiveness (or cost-benefit, cost-utility) |  | ✓ | ✓ | ✓ | ✓ |  |  | 96% | [39] |
|  | Fraser, 2022 | Cost-of-program |  |  | ✓ | ✓ | ✓ |  |  | 100% | [40] |
|  | Tempia, 2019 | Cost-of-illness | ✓ |  |  |  |  |  |  | 100% | [41] |
|  | Tempia, 2020 | Cost-of-illness |  | ✓ | ✓ | ✓ | ✓ |  |  | 100% | [42] |
|  | de Boer, 2018 | Cost-effectiveness (or cost-benefit, cost-utility) | ✓ |  |  |  |  |  |  | 100% | [43] |
| Thailand (UMIC) | Choosakulchart, 2013 | Cost-effectiveness (or cost-benefit, cost-utility) |  |  |  | ✓ |  |  |  | 88% | [44] |
|  | Kittikraisak, 2017 | Cost-effectiveness (or cost-benefit, cost-utility) |  | ✓ |  |  |  |  |  | 100% | [45] |
|  | Kittikraisak, 2018 | Cost-of-illness |  | ✓ |  |  |  |  |  | 92% | [46] |
|  | Meeyai, 2015 | Cost-effectiveness (or cost-benefit, cost-utility) |  | ✓ | ✓ |  |  |  |  | 88% | [47] |
|  | Riewpaiboon, 2021 | Cost-of-program |  |  |  |  | ✓ |  |  | 93% | [48] |
|  | Sribhutorn, 2018 | Cost-effectiveness (or cost-benefit, cost-utility) |  |  |  | ✓ |  |  |  | 96% | [49] |
|  | Suphanchaimat, 2020 | Cost-effectiveness (or cost-benefit, cost-utility) |  |  |  |  |  |  | ✓ | 75% | [50] |
| Turkiye (UMIC) | Akin, 2016 | Cost-effectiveness (or cost-benefit, cost-utility) |  |  |  | ✓ |  |  |  | 100% | [51] |
| Ukraine (LMIC) | Kovacs, 2014 | Cost-of-illness |  |  | ✓ |  |  |  |  | 77% | [27] |
|  | Kyi-Kokarieva, 2021 | Cost-effectiveness (or cost-benefit, cost-utility) |  |  |  |  |  | ✓ |  | 52% | [52] |
| Vietnam (LMIC) | Vo, 2017 | Cost-of-illness | ✓ | ✓ | ✓ |  |  |  |  | 92% | [53] |
|  | Vo, 2017 | Cost-of-illness | ✓ | ✓ | ✓ |  |  |  |  | 92% | [54] |
|  | de Boer, 2018 | Cost-effectiveness (or cost-benefit, cost-utility) | ✓ |  |  |  |  |  |  | 100% | [43] |

Abbreviations: LIC, low-income country; LMIC, lower-middle income country; SAGE, Strategic Advisory Committee of Experts on Immunization; UMIC, upper-middle income country; US$, US Dollars

^1^Although SAGE recommendations specifically reference children aged <5 years [55], publications with data for children aged <18 years were included.

^2^Publications with data for adults aged ≥60 years.

^3^Bangladesh changed classification from LIC to LMIC in 2014 [56], after the study period, and was thus classified as LIC.

^4^China was classified as both UMIC and LMIC corresponding to studies before and after an upward change in World Bank classification in 2010 [56].

^5^Kenya changed classification from LIC to LMIC in 2014 [56], during the study period for both studies, and was thus classified as LMIC for both.

**References**

1. Pallas SW, Ahmeti A, Morgan W, Preza I, Nelaj E, Ebama M, et al. Program cost analysis of influenza vaccination of health care workers in Albania. Vaccine. 2020;38(2):220-7. doi: <https://dx.doi.org/10.1016/j.vaccine.2019.10.027>.

2. Giglio N, Gentile A, Lees L, Micone P, Armoni J, Reygrobellet C, et al. Public health and economic benefits of new pediatric influenza vaccination programs in Argentina. Hum Vaccin Immunother. 2012;8(3):312-22. doi: <https://dx.doi.org/10.4161/hv.18569>.

3. Bhuiyan MU, Luby SP, Alamgir NI, Homaira N, Mamun AA, Khan JA, et al. Economic burden of influenza-associated hospitalizations and outpatient visits in Bangladesh during 2010. Influenza Other Respir Viruses. 2014;8(4):406-13. doi: <https://dx.doi.org/10.1111/irv.12254>.

4. Chen C, Liu GE, Wang MJ, Gao TF, Jia HP, Yang H, et al. Cost-effective analysis of seasonal influenza vaccine in elderly Chinese population [in Chinese]. Zhonghua Yu Fang Yi Xue Za Zhi. 2019;53(10):993-9. doi: <https://dx.doi.org/10.3760/cma.j.issn.0253-9624.2019.10.008>.

5. Gong H, Shen X, Yan H, Lu W, Zhong G, Dong K, et al. Estimating the disease burden of seasonal influenza in China, 2006-2019 [in Chinese]. Zhonghua Yi Xue Za Zhi. 2021;101(8):560-7. doi: <http://dx.doi.org/10.3760/cma.j.cn112137-20201210-03323>.

6. Guo RN, Zheng HZ, Huang LQ, Zhou Y, Zhang X, Liang CK, et al. Epidemiologic and economic burden of influenza in the outpatient setting: a prospective study in a subtropical area of China. PLoS ONE. 2012;7(7):e41403. doi: <https://dx.doi.org/10.1371/journal.pone.0041403>.

7. Jiang M, Li P, Wang W, Zhao M, Atif N, Zhu S, et al. Cost-effectiveness of quadrivalent versus trivalent influenza vaccine for elderly population in China. Vaccine. 2020;38(5):1057-64. doi: <https://dx.doi.org/10.1016/j.vaccine.2019.11.045>.

8. Lai X, Rong H, Ma X, Hou Z, Li S, Jing R, et al. The Economic Burden of Influenza-Like Illness among Children, Chronic Disease Patients, and the Elderly in China: A National Cross-Sectional Survey. Int J Environ Res Public Health. 2021;18(12):10. doi: <https://dx.doi.org/10.3390/ijerph18126277>.

9. Wang D, Zhang T, Wu J, Jiang Y, Ding Y, Hua J, et al. Socio-economic burden of influenza among children younger than 5 years in the outpatient setting in Suzhou, China. PLoS ONE. 2013;8(8):e69035. doi: <https://dx.doi.org/10.1371/journal.pone.0069035>.

10. Wang X, Cai J, Yao W, Zhu Q, Zeng M. Socio-economic impact of influenza in children:a single-centered hospital study in Shanghai [in Chinese]. Zhonghua Liu Xing Bing Xue Za Zhi. 2015;36(1):27-30.

11. Wang SY, Gan ZK, Shao YZ, Chen ZP, Lyu HK. Disease burden of influenza in schools and child care settings in rural areas of Hangzhou, 2016-2018 [in Chinese]. Zhonghua Yu Fang Yi Xue Za Zhi. 2019;53(7):713-8. doi: <https://dx.doi.org/10.3760/cma.j.issn.0253-9624.2019.07.011>.

12. Wang Y, Chen L, Cheng F, Biggerstaff M, Situ S, Zhou S, et al. Economic burden of influenza illness among children under 5 years in Suzhou, China: Report from the cost surveys during 2011/12 to 2016/17 influenza seasons. Vaccine. 2021;39(8):1303-9. doi: <https://dx.doi.org/10.1016/j.vaccine.2020.12.075>.

13. Wu XL, Ye ZJ, Xie F, Huang DF, Kong TJ, Feng SX, et al. Based on a Markov model, cost-effectiveness analysis of influenza vaccination among people aged 60 years and older in Shenzhen [in Chinese]. Zhonghua Liu Xing Bing Xue Za Zhi. 2022;43(7):1140-6. doi: <https://dx.doi.org/10.3760/cma.j.cn112338-20211221-01005>.

14. Yan H, Yang J, Chen Z, Gong H, Zhong G, Yu H. Cost-effectiveness analysis of quadrivalent influenza vaccination for older adults aged 60 and above in mainland China [in Chinese]. Zhonghua Yi Xue Za Zhi. 2021;101(30):2405-12. doi: <https://dx.doi.org/10.3760/cma.j.cn112137-21210123-00224>.

15. Yang J, Jit M, Leung KS, Zheng YM, Feng LZ, Wang LP, et al. The economic burden of influenza-associated outpatient visits and hospitalizations in China: a retrospective survey. Infect Dis Poverty. 2015;4:44. doi: <https://dx.doi.org/10.1186/s40249-015-0077-6>.

16. Yang J, Atkins KE, Feng L, Pang M, Zheng Y, Liu X, et al. Seasonal influenza vaccination in China: Landscape of diverse regional reimbursement policy, and budget impact analysis. Vaccine. 2016;34(47):5724-35. doi: 10.1016/j.vaccine.2016.10.013.

17. Yang J, Yan H, Feng LZ, Yu HJ. Cost-effectiveness of potential government fully-funded influenza vaccination in population with diabetes in China [in Chinese]. Zhonghua Yu Fang Yi Xue Za Zhi. 2019;53(10):1000-6. doi: 10.3760/cma.j.issn.0253-9624.2019.10.009.

18. Yang J, Atkins KE, Feng L, Baguelin M, Wu P, Yan H, et al. Cost-effectiveness of introducing national seasonal influenza vaccination for adults aged 60 years and above in mainland China: a modelling analysis. BMC Med. 2020;18(1):90. doi: <https://dx.doi.org/10.1186/s12916-020-01545-6>.

19. Yu J, Zhang T, Wang Y, Gao JM, Hua J, Tian JM, et al. Clinical characteristics and economic burden of influenza among children under 5 years old, in Suzhou, 2011-2017 [in Chinese]. Zhonghua Liu Xing Bing Xue Za Zhi. 2018;39(6):847-51. doi: 10.3760/cma.j.issn.0254-6450.2018.06.029.

20. Zhang X, Zhang J, Chen L, Feng L, Yu H, Zhao G, et al. Pneumonia and influenza hospitalizations among children under 5 years of age in Suzhou, China, 2005-2011. Influenza Other Respir Viruses. 2017;11(1):15-22. doi: <https://dx.doi.org/10.1111/irv.12405>.

21. Zhou L, Situ S, Feng Z, Atkins CY, Fung IC, Xu Z, et al. Cost-effectiveness of alternative strategies for annual influenza vaccination among children aged 6 months to 14 years in four provinces in China. PLoS ONE. 2014;9(1):e87590. doi: <https://dx.doi.org/10.1371/journal.pone.0087590>.

22. Castillo-Rodriguez L, Malo-Sanchez D, Diaz-Jimenez D, Garcia-Velasquez I, Pulido P, Castaneda-Orjuela C. Economic costs of severe seasonal influenza in Colombia, 2017-2019: A multi-center analysis. PLoS ONE. 2022;17(6):e0270086. doi: <https://dx.doi.org/10.1371/journal.pone.0270086>.

23. Lara C, De Graeve D, Franco F. Cost-Effectiveness Analysis of Pneumococcal and Influenza Vaccines Administered to Children Less Than 5 Years of Age in a Low-Income District of Bogota, Colombia. Value Health Reg Issues. 2018;17:21-31. doi: <https://dx.doi.org/10.1016/j.vhri.2018.01.001>.

24. Salcedo-Mejia F, Alvis-Zakzuk NJ, Carrasquilla-Sotomayor M, Redondo HP, Castaneda-Orjuela C, De la Hoz-Restrepo F, et al. Economic Cost of Severe Acute Respiratory Infection Associated to Influenza in Colombian Children: A Single Setting Analysis. Value Health Reg Issues. 2019;20:159-63. doi: <https://dx.doi.org/10.1016/j.vhri.2019.07.010>.

25. Jara JH, Azziz-Baumgartner E, De Leon T, Luciani K, Brizuela YS, Estripeaut D, et al. Costs associated with acute respiratory illness and select virus infections in hospitalized children, El Salvador and Panama, 2012-2013. J Infect. 2019;79(2):108-14. doi: 10.1016/j.jinf.2019.05.021.

26. Koul PA, Bhavsar A, Mir H, Simmerman M, Khanna H. Epidemiology and costs of severe acute respiratory infection and influenza hospitalizations in adults with diabetes in India. J Infect Dev Ctries. 2019;13(3):204-11. doi: <https://dx.doi.org/10.3855/jidc.10903>.

27. Kovacs G, Kalo Z, Jahnz-Rozyk K, Kyncl J, Csohan A, Pistol A, et al. Medical and economic burden of influenza in the elderly population in central and eastern European countries. Hum Vaccin Immunother. 2014;10(2):428-40. doi: <https://dx.doi.org/10.4161/hv.26886>.

28. Dawa J, Emukule GO, Barasa E, Widdowson MA, Anzala O, van Leeuwen E, et al. Seasonal influenza vaccination in Kenya: an economic evaluation using dynamic transmission modelling. BMC Med. 2020;18(1):223. doi: <https://dx.doi.org/10.1186/s12916-020-01687-7>.

29. Emukule GO, Ndegwa LK, Washington ML, Paget JW, Duque J, Chaves SS, et al. The cost of influenza-associated hospitalizations and outpatient visits in Kenya. BMC Public Health. 2019;19(Suppl 3):471. doi: <https://dx.doi.org/10.1186/s12889-019-6773-6>.

30. Ortega-Sanchez IR, Mott JA, Kittikraisak W, Khanthamaly V, McCarron M, Keokhonenang S, et al. Cost-effectiveness of seasonal influenza vaccination in pregnant women, healthcare workers and adults >= 60 years of age in Lao People's Democratic Republic. Vaccine. 2021;39(52):7633-45. doi: <https://dx.doi.org/10.1016/j.vaccine.2021.11.011>.

31. Pecenka C, Munthali S, Chunga P, Levin A, Morgan W, Lambach P, et al. Maternal influenza immunization in Malawi: Piloting a maternal influenza immunization program costing tool by examining a prospective program. PLoS ONE. 2017;12(12):e0190006. doi: <https://dx.doi.org/10.1371/journal.pone.0190006>.

32. Tohiar MAH, Jaafar S, Aizuddin AN, Leong TK, Abdul Rahim AS. Workplace influenza vaccination in private hospital setting: a cost-benefit analysis. Ann Occup Environ Med. 2022;34:e3. doi: <https://dx.doi.org/10.35371/aoem.2022.34.e3>.

33. Orenstein EW, Orenstein LA, Diarra K, Djiteye M, Sidibe D, Haidara FC, et al. Cost-effectiveness of maternal influenza immunization in Bamako, Mali: A decision analysis. PLoS ONE. 2017;12(2):e0171499. doi: <https://dx.doi.org/10.1371/journal.pone.0171499>.

34. Betancourt-Cravioto M, Falcon-Lezama JA, Saucedo-Martinez R, Alfaro-Cortes MM, Tapia-Conyer R. Public Health and Economic Benefits of Influenza Vaccination of the Population Aged 50 to 59 Years without Risk Factors for Influenza Complications in Mexico: A Cross-Sectional Epidemiological Study. Vaccines (Basel). 2021;9(3):24. doi: <https://dx.doi.org/10.3390/vaccines9030188>.

35. Falcon-Lezama JA, Saucedo-Martinez R, Betancourt-Cravioto M, Alfaro-Cortes MM, Bahena-Gonzalez RI, Tapia-Conyer R. Influenza in the school-aged population in Mexico: burden of disease and cost-effectiveness of vaccination in children. BMC Infect Dis. 2020;20(1):240. doi: <https://dx.doi.org/10.1186/s12879-020-4948-5>.

36. Reyes-Lopez A, Moreno-Espinosa S, Hernandez-Olivares YO, Rodolfo Norberto JJ. Economic issues of Severe Acute Respiratory Infections for influenza in Mexican children attended in a tertiary public hospital. PLoS ONE. 2022;17(9):e0273923. doi: <https://dx.doi.org/10.1371/journal.pone.0273923>.

37. Tapia-Conyer R, Betancourt-Cravioto M, Montoya A, Falcon-Lezama JA, Alfaro-Cortes MM, Saucedo-Martinez R. A Call for a Reform of the Influenza Immunization Program in Mexico: Epidemiologic and Economic Evidence for Decision Making. Vaccines (Basel). 2021;9(3):19. doi: <https://dx.doi.org/10.3390/vaccines9030286>.

38. Biggerstaff M, Cohen C, Reed C, Tempia S, McMorrow ML, Walaza S, et al. A cost-effectiveness analysis of antenatal influenza vaccination among HIV-infected and HIV-uninfected pregnant women in South Africa. Vaccine. 2019;37(46):6874-84. doi: <https://dx.doi.org/10.1016/j.vaccine.2019.09.059>.

39. Edoka I, Kohli-Lynch C, Fraser H, Hofman K, Tempia S, McMorrow M, et al. A cost-effectiveness analysis of South Africa's seasonal influenza vaccination programme. Vaccine. 2021;39(2):412-22. doi: <https://dx.doi.org/10.1016/j.vaccine.2020.11.028>.

40. Fraser H, Tombe-Mdewa W, Kohli-Lynch C, Hofman K, Tempia S, McMorrow M, et al. Costs of seasonal influenza vaccination in South Africa. Influenza Other Respir Viruses. 2022;16(5):873-80. doi: <https://dx.doi.org/10.1111/irv.12987>.

41. Tempia S, Moyes J, Cohen AL, Walaza S, Edoka I, McMorrow ML, et al. Health and economic burden of influenza-associated illness in South Africa, 2013-2015. Influenza Other Respir Viruses. 2019;13(5):484-95. doi: <https://dx.doi.org/10.1111/irv.12650>.

42. Tempia S, Moyes J, Cohen AL, Walaza S, McMorrow ML, Edoka I, et al. Influenza economic burden among potential target risk groups for immunization in South Africa, 2013-2015. Vaccine. 2020;38(45):7007-14. doi: <https://dx.doi.org/10.1016/j.vaccine.2020.09.033>.

43. de Boer PT, Kelso JK, Halder N, Nguyen TP, Moyes J, Cohen C, et al. The cost-effectiveness of trivalent and quadrivalent influenza vaccination in communities in South Africa, Vietnam and Australia. Vaccine. 2018;36(7):997-1007. doi: <https://dx.doi.org/10.1016/j.vaccine.2017.12.073>.

44. Choosakulchart P, Kittisopee T, Takdhada S, Lubell Y, Robinson J. Cost-utility evaluation of influenza vaccination in patients with existing coronary heart diseases in Thailand. Asian Biomedicine. 2013;7(3):425-35. doi: <http://dx.doi.org/10.5372/1905-7415.0703.196>.

45. Kittikraisak W, Suntarattiwong P, Ditsungnoen D, Pallas SE, Abimbola TO, Klungthong C, et al. Cost-effectiveness of inactivated seasonal influenza vaccination in a cohort of Thai children 60 months of age. PLoS One. 2017;12(8). doi: <http://dx.doi.org/10.1371/journal.pone.0183391>.

46. Kittikraisak W, Suntarattiwong P, Kanjanapattanakul W, Ditsungnoen D, Klungthong C, Lindblade KA, et al. Comparison of incidence and cost of influenza between healthy and high-risk children <60 months old in Thailand, 2011-2015. PLoS ONE. 2018;13(5):e0197207. doi: <https://dx.doi.org/10.1371/journal.pone.0197207>.

47. Meeyai A, Praditsitthikorn N, Kotirum S, Kulpeng W, Putthasri W, Cooper BS, et al. Seasonal influenza vaccination for children in Thailand: a cost-effectiveness analysis. PLoS Med. 2015;12(5):e1001829. doi: <https://dx.doi.org/10.1371/journal.pmed.1001829>.

48. Riewpaiboon A. Cost analysis of influenza vaccination for pregnant women in Thailand. Pharmaceutical Sciences Asia. 2021;48(2):99-106. doi: <http://dx.doi.org/10.29090/psa.2021.02.19.063>.

49. Sribhutorn A, Phrommintikul A, Wongcharoen W, Chaikledkaew U, Eakanunkul S, Sukonthasarn A. Influenza vaccination in acute coronary syndromes patients in Thailand: the cost-effectiveness analysis of the prevention for cardiovascular events and pneumonia. J Geriatr Cardiol. 2018;15(6):413-21. doi: <https://dx.doi.org/10.11909/j.issn.1671-5411.2018.06.008>.

50. Suphanchaimat R, Doung-Ngern P, Ploddi K, Suthachana S, Phaiyarom M, Pachanee K, et al. Cost Effectiveness and Budget Impact Analyses of Influenza Vaccination for Prisoners in Thailand: An Application of System Dynamic Modelling. Int J Environ Res Public Health. 2020;17(4):14. doi: <https://dx.doi.org/10.3390/ijerph17041247>.

51. Akin L, Macabeo B, Caliskan Z, Altinel S, Satman I. Cost-Effectiveness of Increasing Influenza Vaccination Coverage in Adults with Type 2 Diabetes in Turkey. PLoS ONE. 2016;11(6):e0157657. doi: <https://dx.doi.org/10.1371/journal.pone.0157657>.

52. Kyi-Kokarieva VG, Padalkо LI, Kriachkova LV. Socio-economic substantiation of expediency of seasonal influenza vaccine prophylaxis among medical workers. Medicni Perspektivi. 2021;26(4):205-12. doi: 10.26641/2307-0404.2021.4.248235.

53. Vo TQ, Chaikledkaew U, Hoang MV, Riewpaiboon A. Economic burden of influenza at a tertiary hospital in Vietnam. Asian Pac J Trop Dis. 2017;7(3):144-50. doi: <http://dx.doi.org/10.12980/apjtd.7.2017D6-348>.

54. Vo TQ, Chaikledkaew U, Van Hoang M, Riewpaiboon A. Social and economic burden of patients with influenza-like illness and clinically diagnosed flu treated at various health facilities in Vietnam. Clinicoecon Outcomes Res. 2017;9:423-32. doi: <https://dx.doi.org/10.2147/CEOR.S131687>.

55. World Health Organization. Vaccines against influenza: WHO position paper—May 2022. Wkly Epidemiol Rec. 2022;19:185-208.

56. The World Bank Group. World Bank Open Data 2023 [accessed 2022 November 1]. Available from: <https://data.worldbank.org/>.
