# Supplementary material for: Costs and cost-effectiveness of influenza illness and vaccination in low- and middle-income countries: A systematic review from 2012 to 2022
Source: PLoS Med. 2024 Jan 5;21(1):e1004333. doi: 10.1371/journal.pmed.1004333 (PMC10802964; doi:10.1371/journal.pmed.1004333)
Supplement: S4 Table — HIV, human immunodeficiency virus; ILI, influenza-like illness; LCI, laboratory-confirmed influenza; LIC, low-income country; LMIC, lower-middle income country; NA, not applicable; NR, not reported; P&I, pneumonia and influenza hospitalization; SARI, severe acute respiratory infection; TB, tuberculosis; UMC, underlying medical condition; UMIC, upper-middle income country; US$, US Dollars. 1Median costs were preferentially abstracted from source publications; if unavailable, mean costs were abstracted. 2No cost-of-illness papers were identified for healthcare workers or individuals in congregate living settings in low- and middle-income countries. 3For source publications presenting results for both LCI and syndromic illness, the results for LCI were used. 4Direct costs were all medical and non-medical costs directly attributable to patient care. 5Calculated or converted value; not presented in source publication. 6Indirect costs were all costs not directly attributable to patient care (e.g., lost earnings or lost productivity). 7Direct medical and non-medical costs were summarized separately in the source publication; medians were summed to obtain a total median direct cost. 8China changed classification from LMIC to UMIC in 2010 [24], after the study period, and was thus classified as LMIC for this study. 9No indirect costs were included in the total estimate because of study perspective. The specific payer was not specified in the source publication. 10Kenya changed classification from LIC to LMIC in 2014 [24], during the study period, and was thus classified as LMIC. 11Bangladesh changed classification from LIC to LMIC in 2014 [24], after the study period, and was thus classified as LIC. 12Included only direct medical costs (no non-medical costs). 13Although SAGE recommendations specifically reference children aged <5 years [1], publications with data for children aged <18 years were included. 14The full publication study period was 2005–2011; however, 2009–2010 [file pmed.1004333.s007.docx]

**Table S4: Costs of influenza illness^1^, by Strategic Advisory Committee of Experts on Immunization (SAGE) target group^2^ and disease severity (outpatient vs. hospitalized), in low- and middle-income countries**

| **Study characteristics** | | | | | | | | **Outpatient visits** | | | **Hospitalizations** | | | |
| --- | --- | --- | --- | --- | --- | --- | --- | --- | --- | --- | --- | --- | --- | --- |
| **Income group** | **Study** | **Country** | **Target group details** | **Data source; public or private facilities** | **Illness case definition^3^** | **Study period** | **Perspective** | **Direct^4^ cost per episode (2022 US$)^5^** | **Indirect^6^ cost per episode (2022 US$)^5^** | **Total cost per episode (2022 US$)^5^** | **Direct^4^ cost per episode (2022 US$)^5^** | **Indirect^6^ cost per episode (2022 US$)^5^** | **Total cost per episode (2022 US$)^5^** | **Median length of hospitalization (days)** |
| **General population** | | | | | | | | | | | | | | |
| UMIC | Gong, 2021 [1] | China | All ages | NR | ILI/SARI | 2006–19 | Societal | $107.13 | $50.75 | $149.98 | $1392.72 | $193.97 | $1617.14 | -- |
| UMIC | Yang, 2015 [2] | China | All ages | 554 facilities; NR | LCI | 2013–14 | Societal | $77.35^7^ | $52.79 | $155.92 | $1193.36^7^ | $181.70 | $1416.80 | 9 |
| UMIC | Tempia, 2019 [3] | South Africa | All ages | 7 hospitals and 2 clinics; public | ILI/SARI | 2013–15 | Societal | $32.20 | $15.50 | $47.71 | $874.24 | $42.94 | $918.37 | 5.3 |
| UMIC | Castillo-Rodriguez, 2022 [4] | Colombia | All ages | 6 hospitals; NR | LCI | 2017–19 | Societal | -- | -- | -- | $795.11 | $166.97 | $962.09 | 7 |
| LMIC^8^ | Guo, 2012 [5] | China | All ages | 28 hospitals and clinics; public | ILI | 2008–09 | Payer^9^ | $29.80 | -- | -- | -- | -- | -- | -- |
| LMIC^10^ | Emukule, 2019 [6] | Kenya | All ages | 4 hospitals and 1 clinic; public and private | LCI | 2013–14 | Societal | $9.26 | $15.49 | $23.91 | $90.99 | $50.67 | $142.17 | 4 |
| LMIC | Vo, 2017 [7] | Vietnam | All ages | 1 hospital; NR | SARI | 2013–15 | Healthcare system | -- | -- | -- | $134.27^12^ | -- | -- | 7 |
| LMIC | Vo, 2017 [8] | Vietnam | All ages | 15 pharmacies, 3 clinics, and 1 hospital; private | ILI/SARI | 2016 | Societal | $104.46 | $21.63 | $128.35 | $99.31 | $21.63 | $110.55 | -- |
| LIC^11^ | Bhuiyan, 2014 [9] | Bangladesh | All ages | 4 hospitals; public and private | LCI | 2010 | Societal | $5.47 | $11.83 | $6.24 | $77.67 | $28.92 | $106.85 | 3 |
| **Children**^13^ | | | | | | | | | | | | | | |
| UMIC | Zhang, 2017 [10] | China | <5 years | 9 hospitals; NR | P&I | 2005–09^14^ | Payer^9^ | -- | -- | -- | $480.97^12^ | -- | -- | 7.1 |
| UMIC | Wang, 2013 [11] | China | <5 years | 1 hospital; NR | LCI | 2011–12 | Societal | $110.28 | $48.39 | $159.04 | -- | -- | -- | -- |
| UMIC | Wang, 2015 [12] | China | 6 months–11.5 years | 1 hospital; NR | LCI | 2011–12 | Societal | $139.91 | $58.22 | $198.13 | -- | -- | -- | -- |
| UMIC | Kittikraisak, 2018 [13] | Thailand | <5 years | 1 hospital; public | LCI | 2011–15 | Societal | -- | -- | $26.24 | -- | -- | $293.40 | 10 |
| UMIC | Yu, 2018 [14] | China | <5 years | 1 hospital; NR | LCI | 2011–17 | Patient | $107.86 | $23.94 | $131.80 | -- | -- | -- | -- |
| UMIC | Wang, 2021 [15] | China | <5 years | 1 hospital; NR | LCI | 2011–17 | Societal | $118.98 | $67.85 | $186.83 | $1288.85 | $365.42 | $1654.23 | 7 |
| UMIC | Jara, 2019 [16] | Panama | <10 years | 2 hospitals; public | LCI | 2012–13 | Societal | -- | -- | -- | -- | -- | $554.32 | -- |
| UMIC | Yang, 2015 [2] | China | <5 years | 554 facilities; NR | LCI | 2013–14 | Societal | $95.76^7^ | $69.98 | $192.75 | $1257.20^7^ | $192.75 | $1474.51 | -- |
| UMIC | Tempia, 2020 [17] | South Africa | 6–59 months | 7 hospitals and 2 clinics; public | ILI/SARI | 2013–15 | Societal | $33.40 | $17.89 | $50.09 | $743.04 | $21.47 | $764.51 | -- |
| UMIC | Reyes-Lopez, 2022 [18] | Mexico | Maximum age NR | 1 hospital; public | LCI | 2013–18 | Payer^9^ |  |  |  | $10631.53 |  |  | 6.7 |
| UMIC | Salcedo-Mejia, 2019 [19] | Colombia | <18 years | 1 hospital; NR | LCI | 2014 | Societal | -- | -- | -- | $1987.30 | $99.03 | $2202.74 | 8.9 |
| UMIC | Wang, 2019 [20] | China | 3–17 years | NR | ILI/SARI | 2016–18 | Societal | $35.10 | $4.55 | $41.75 | $471.46 | $69.97 | $524.62 | -- |
| UMIC | Lai, 2021 [21] | China | 6–59 months | 148 community health centers; public | ILI/SARI | 2019 | Societal | $102.18 | -- | -- | $82.99 | -- | -- | -- |
| LMIC | Jara, 2019 [16] | El Salvador | <10 years | 3 hospitals; public | LCI | 2012–13 | Societal | -- | -- | -- | -- | -- | $176.30 | -- |
| LMIC^10^ | Emukule, 2019 [6] | Kenya | <5 years | 4 hospitals and 1 clinic; public and private | LCI | 2013–14 | Societal | $10.40 | $16.73 | $25.92 | $90.98 | $46.97 | $137.81 | 4 |
| LMIC | Vo, 2017 [7] | Vietnam | ≤14 years | 1 hospital; NR | SARI | 2013–15 | Healthcare system | -- | -- | -- | $96.15^12^ | -- | -- | -- |
| **Older adults** | | | | | | | | | | | | | | |
| UMIC | Kovacs, 2014 [22] | Kazakhstan | ≥65 years | 19 hospitals; NR | ILI/SARI | 2011–12 | Payer^9^ | $19.49 (medical only) | -- | -- | $302.24^12^ | -- | -- | -- |
| UMIC | Kovacs, 2014 [22] | Romania | ≥65 years | 26 hospitals; NR | ILI/SARI | 2011–12 | Payer^9^ | $26.93 (medical only) | -- | -- | $1048.11^12^ | -- | -- | -- |
| LMIC | Kovacs, 2014 [22] | Ukraine | ≥65 years | 10 hospitals; NR | ILI/SARI | 2011–12 | Payer^9^ | $26.22 (medical only) | -- | -- | $110.73^12^ | -- | -- | -- |
| UMIC | Yang, 2015 [2] | China | ≥60 years | 554 facilities; NR | LCI | 2013–14 | Societal | $66.30^7^ | $45.43 | $164.52 | $2499.66^7^ | $236.95 | $2729.25 | -- |
| LMIC | Vo, 2017 [7] | Vietnam | >64 years | 1 hospital; NR | SARI | 2013–15 | Healthcare system | -- | -- | -- | $282.37^12^ | -- | -- | -- |
| UMIC | Tempia, 2020 [17] | South Africa | ≥65 years | 7 hospitals and 2 clinics; public | ILI/SARI | 2013–15 | Societal | $32.20 | $5.96 | $38.17 | $1056.72 | $2.39 | $1059.10 | -- |
| UMIC | Lai, 2021 [21] | China | ≥60 years | 148 community health centers; public | ILI/SARI | 2019 | Societal | $60.93 | -- | -- | $167.08 | -- | -- | -- |
| **Persons with chronic medical conditions** | | | | | | | | | | | | | | |
| UMIC | Yang, 2015 [2] | China | All ages with UMC | 554 facilities; NR | LCI | 2013–14 | Societal | $99.45^7^ | $54.02 | $176.79 | $1357.87^7^ | $213.63 | $1578.86 | -- |
| UMIC | Tempia, 2020 [17] | South Africa | 5–64 years with HIV, TB, or other UMC | 7 hospitals and 2 clinics; public | ILI/SARI | 2013–15 | Societal | $32.20 | $11.93 | $44.13 | $1084.15 | $63.21 | $1147.36 | -- |
| UMIC | Lai, 2021 [21] | China | 18–59 years with UMC | 148 community health centers; public | ILI/SARI | 2019 | Societal | $37.92 | -- | -- | $62.52 | -- | -- | -- |
| LMIC | Koul, 2019 [23] | India | ≥18 years with diabetes | 1 hospital; public | LCI | 2015–17 | Societal | -- | -- | -- | -- | -- | $847.60 | 9 |
| **Pregnant persons** | | | | | | | | | | | | | | |
| UMIC | Tempia, 2020 [17] | South Africa | NA | 7 hospitals and 2 clinics; public | ILI/SARI | 2013–15 | Societal | $31.01 | $5.96 | $36.97 | $1024.52 | $63.21 | $1088.92 | -- |
| LIC | Orenstein, 2017 [24] | Mali | NA | 6 community and referral health centers | LCI and ILI^15^ | 2011–14 | Societal |  |  | $5.45 (LCI)^15^ |  |  | $189.98 (ILI)^15^ |  |

Abbreviations: HIV, human immunodeficiency virus; ILI, influenza-like illness; LCI, laboratory-confirmed influenza; LIC, low-income country; LMIC, lower-middle income country; NA, not applicable; NR, not reported; P&I, pneumonia and influenza hospitalization; SARI, severe acute respiratory infection; TB, tuberculosis; UMC, underlying medical condition; UMIC, upper-middle income country; US$, US Dollars

^1^Median costs were preferentially abstracted from source publications; if unavailable, mean costs were abstracted.

^2^No cost-of-illness papers were identified for healthcare workers or individuals in congregate living settings in low- and middle-income countries.

^3^For source publications presenting results for both LCI and syndromic illness, the results for LCI were used.

^4^Direct costs were all medical and non-medical costs directly attributable to patient care.

^5^Calculated or converted value; not presented in source publication.

^6^Indirect costs were all costs not directly attributable to patient care (e.g., lost earnings or lost productivity).

^7^Direct medical and non-medical costs were summarized separately in the source publication; medians were summed to obtain a total median direct cost.

^8^China changed classification from LMIC to UMIC in 2010 [25], after the study period, and was thus classified as LMIC for this study.

^9^No indirect costs were included in the total estimate because of study perspective. The specific payer was not specified in the source publication.

^10^Kenya changed classification from LIC to LMIC in 2014 [25], during the study period, and was thus classified as LMIC.

^11^Bangladesh changed classification from LIC to LMIC in 2014 [25], after the study period, and was thus classified as LIC.

^12^Included only direct medical costs (no non-medical costs).

^13^Although SAGE recommendations specifically reference children aged <5 years [26], publications with data for children aged <18 years were included.

^14^The full publication study period was 2005–2011; however, 2009–2010 and 2010–2011 were excluded because of H1N1 pandemic activity. Abstracted values represent the median of 2005–2009 annual values.

^15^Cost data were only available for one hospitalized LCI case; thus, ILI hospitalization costs were abstracted.

**References**

1. Gong H, Shen X, Yan H, Lu W, Zhong G, Dong K, et al. Estimating the disease burden of seasonal influenza in China, 2006-2019 [in Chinese]. Zhonghua Yi Xue Za Zhi. 2021;101(8):560-7. doi: <http://dx.doi.org/10.3760/cma.j.cn112137-20201210-03323>.

2. Yang J, Jit M, Leung KS, Zheng YM, Feng LZ, Wang LP, et al. The economic burden of influenza-associated outpatient visits and hospitalizations in China: a retrospective survey. Infect Dis Poverty. 2015;4:44. doi: <https://dx.doi.org/10.1186/s40249-015-0077-6>.

3. Tempia S, Moyes J, Cohen AL, Walaza S, Edoka I, McMorrow ML, et al. Health and economic burden of influenza-associated illness in South Africa, 2013-2015. Influenza Other Respir Viruses. 2019;13(5):484-95. doi: <https://dx.doi.org/10.1111/irv.12650>.

4. Castillo-Rodriguez L, Malo-Sanchez D, Diaz-Jimenez D, Garcia-Velasquez I, Pulido P, Castaneda-Orjuela C. Economic costs of severe seasonal influenza in Colombia, 2017-2019: A multi-center analysis. PLoS ONE. 2022;17(6):e0270086. doi: <https://dx.doi.org/10.1371/journal.pone.0270086>.

5. Guo RN, Zheng HZ, Huang LQ, Zhou Y, Zhang X, Liang CK, et al. Epidemiologic and economic burden of influenza in the outpatient setting: a prospective study in a subtropical area of China. PLoS ONE. 2012;7(7):e41403. doi: <https://dx.doi.org/10.1371/journal.pone.0041403>.

6. Emukule GO, Ndegwa LK, Washington ML, Paget JW, Duque J, Chaves SS, et al. The cost of influenza-associated hospitalizations and outpatient visits in Kenya. BMC Public Health. 2019;19(Suppl 3):471. doi: <https://dx.doi.org/10.1186/s12889-019-6773-6>.

7. Vo TQ, Chaikledkaew U, Hoang MV, Riewpaiboon A. Economic burden of influenza at a tertiary hospital in Vietnam. Asian Pac J Trop Dis. 2017;7(3):144-50. doi: <http://dx.doi.org/10.12980/apjtd.7.2017D6-348>.

8. Vo TQ, Chaikledkaew U, Van Hoang M, Riewpaiboon A. Social and economic burden of patients with influenza-like illness and clinically diagnosed flu treated at various health facilities in Vietnam. Clinicoecon Outcomes Res. 2017;9:423-32. doi: <https://dx.doi.org/10.2147/CEOR.S131687>.

9. Bhuiyan MU, Luby SP, Alamgir NI, Homaira N, Mamun AA, Khan JA, et al. Economic burden of influenza-associated hospitalizations and outpatient visits in Bangladesh during 2010. Influenza Other Respir Viruses. 2014;8(4):406-13. doi: <https://dx.doi.org/10.1111/irv.12254>.

10. Zhang X, Zhang J, Chen L, Feng L, Yu H, Zhao G, et al. Pneumonia and influenza hospitalizations among children under 5 years of age in Suzhou, China, 2005-2011. Influenza Other Respir Viruses. 2017;11(1):15-22. doi: <https://dx.doi.org/10.1111/irv.12405>.

11. Wang D, Zhang T, Wu J, Jiang Y, Ding Y, Hua J, et al. Socio-economic burden of influenza among children younger than 5 years in the outpatient setting in Suzhou, China. PLoS ONE. 2013;8(8):e69035. doi: <https://dx.doi.org/10.1371/journal.pone.0069035>.

12. Wang X, Cai J, Yao W, Zhu Q, Zeng M. Socio-economic impact of influenza in children:a single-centered hospital study in Shanghai [in Chinese]. Zhonghua Liu Xing Bing Xue Za Zhi. 2015;36(1):27-30.

13. Kittikraisak W, Suntarattiwong P, Kanjanapattanakul W, Ditsungnoen D, Klungthong C, Lindblade KA, et al. Comparison of incidence and cost of influenza between healthy and high-risk children <60 months old in Thailand, 2011-2015. PLoS ONE. 2018;13(5):e0197207. doi: <https://dx.doi.org/10.1371/journal.pone.0197207>.

14. Yu J, Zhang T, Wang Y, Gao JM, Hua J, Tian JM, et al. Clinical characteristics and economic burden of influenza among children under 5 years old, in Suzhou, 2011-2017 [in Chinese]. Zhonghua Liu Xing Bing Xue Za Zhi. 2018;39(6):847-51. doi: 10.3760/cma.j.issn.0254-6450.2018.06.029.

15. Wang Y, Chen L, Cheng F, Biggerstaff M, Situ S, Zhou S, et al. Economic burden of influenza illness among children under 5 years in Suzhou, China: Report from the cost surveys during 2011/12 to 2016/17 influenza seasons. Vaccine. 2021;39(8):1303-9. doi: <https://dx.doi.org/10.1016/j.vaccine.2020.12.075>.

16. Jara JH, Azziz-Baumgartner E, De Leon T, Luciani K, Brizuela YS, Estripeaut D, et al. Costs associated with acute respiratory illness and select virus infections in hospitalized children, El Salvador and Panama, 2012-2013. J Infect. 2019;79(2):108-14. doi: 10.1016/j.jinf.2019.05.021.

17. Tempia S, Moyes J, Cohen AL, Walaza S, McMorrow ML, Edoka I, et al. Influenza economic burden among potential target risk groups for immunization in South Africa, 2013-2015. Vaccine. 2020;38(45):7007-14. doi: <https://dx.doi.org/10.1016/j.vaccine.2020.09.033>.

18. Reyes-Lopez A, Moreno-Espinosa S, Hernandez-Olivares YO, Rodolfo Norberto JJ. Economic issues of Severe Acute Respiratory Infections for influenza in Mexican children attended in a tertiary public hospital. PLoS ONE. 2022;17(9):e0273923. doi: <https://dx.doi.org/10.1371/journal.pone.0273923>.

19. Salcedo-Mejia F, Alvis-Zakzuk NJ, Carrasquilla-Sotomayor M, Redondo HP, Castaneda-Orjuela C, De la Hoz-Restrepo F, et al. Economic Cost of Severe Acute Respiratory Infection Associated to Influenza in Colombian Children: A Single Setting Analysis. Value Health Reg Issues. 2019;20:159-63. doi: <https://dx.doi.org/10.1016/j.vhri.2019.07.010>.

20. Wang SY, Gan ZK, Shao YZ, Chen ZP, Lyu HK. Disease burden of influenza in schools and child care settings in rural areas of Hangzhou, 2016-2018 [in Chinese]. Zhonghua Yu Fang Yi Xue Za Zhi. 2019;53(7):713-8. doi: <https://dx.doi.org/10.3760/cma.j.issn.0253-9624.2019.07.011>.

21. Lai X, Rong H, Ma X, Hou Z, Li S, Jing R, et al. The Economic Burden of Influenza-Like Illness among Children, Chronic Disease Patients, and the Elderly in China: A National Cross-Sectional Survey. Int J Environ Res Public Health. 2021;18(12):10. doi: <https://dx.doi.org/10.3390/ijerph18126277>.

22. Kovacs G, Kalo Z, Jahnz-Rozyk K, Kyncl J, Csohan A, Pistol A, et al. Medical and economic burden of influenza in the elderly population in central and eastern European countries. Hum Vaccin Immunother. 2014;10(2):428-40. doi: <https://dx.doi.org/10.4161/hv.26886>.

23. Koul PA, Bhavsar A, Mir H, Simmerman M, Khanna H. Epidemiology and costs of severe acute respiratory infection and influenza hospitalizations in adults with diabetes in India. J Infect Dev Ctries. 2019;13(3):204-11. doi: <https://dx.doi.org/10.3855/jidc.10903>.

24. Orenstein EW, Orenstein LA, Diarra K, Djiteye M, Sidibe D, Haidara FC, et al. Cost-effectiveness of maternal influenza immunization in Bamako, Mali: A decision analysis. PLoS ONE. 2017;12(2):e0171499. doi: <https://dx.doi.org/10.1371/journal.pone.0171499>.

25. The World Bank Group. World Bank Open Data 2023 [accessed 2022 November 1]. Available from: <https://data.worldbank.org/>.

26. World Health Organization. Vaccines against influenza: WHO position paper—May 2022. Wkly Epidemiol Rec. 2022;19:185-208.
