# Supplementary material for: Costs and cost-effectiveness of influenza illness and vaccination in low- and middle-income countries: A systematic review from 2012 to 2022
Source: PLoS Med. 2024 Jan 5;21(1):e1004333. doi: 10.1371/journal.pmed.1004333 (PMC10802964; doi:10.1371/journal.pmed.1004333)
Supplement: S5 Table — DALY, disability-adjusted life year; ICER, incremental cost-effectiveness ratio; Govt, government; HIV, human immunodeficiency virus; LAIV, live attenuated influenza vaccine; LIC, low-income country; LMIC, lower-middle income country; NA, not applicable; NR, not reported; QALY, quality-adjusted life year; QIV, quadrivalent influenza vaccine; TIV, trivalent influenza vaccine; UMIC, upper-middle income country; US$, US Dollars; WTP, willingness-to-pay. 1Cost-effectiveness, cost-utility, and cost-benefit analyses were eligible for inclusion if they included a comparison of influenza vaccination vs. either no vaccination or modifications to current vaccination program. Studies that only compared the cost-effectiveness of different influenza vaccine products were not included. 2Data for each base-scenario intervention or each perspective assessed are presented in individual rows. Sensitivity analyses are not presented. Vaccine coverage was rounded to the nearest integer. 3Calculated or converted value; not presented in source publication. Ranges represent annual seasonal estimates or varying illness attack rate. 4Interpretation per source publication. Interpretations of highly cost-effective and cost-effective were both combined as “cost-effective.” 5The study authors intentionally did not specify a cost-effectiveness threshold or interpretation because a country-specific threshold was not available. Instead, cost-effectiveness acceptability curves were presented over a range of willingness-to-pay thresholds. 6Similar data sources and analysis methods used in both publications; these were counted collectively as one study for Fig 3. 7Interpretation of net costs of vaccination (including illness averted) vs. no vaccination. 8Although SAGE recommendations specifically reference children aged <5 years [1], publications with data for children aged <18 years were included. 9This study also modeled alternative strategies to increase vaccination rates. 10This study used a cost- [file pmed.1004333.s008.docx]

**Table S5: Cost-effectiveness of influenza vaccination^1^, by Strategic Advisory Committee of Experts on Immunization (SAGE) target group, in low- and middle-income countries**

| **Study characteristics** | | | | | | | **Study results** | | | |
| --- | --- | --- | --- | --- | --- | --- | --- | --- | --- | --- |
| **Income group** | **Study** | **Country** | **Target group details** | **Intervention^2^** | **Comparator** | **Perspective** | **ICER (in 2022 US$)^3^** | **Interpretation^4^** | **Cost-effectiveness threshold (in 2022 US$)** | **Cost-effectiveness threshold explanation** |
| **General population** | | | | | | | | | | |
| UMIC | de Boer, 2018 [1] | South Africa | All ages | Vaccination with TIV at 15% coverage | No vaccination | Societal | $1297.71– 2213.60 per QALY | None^5^ | No threshold^5^ | No threshold^5^ |
| UMIC | Betancourt-Craviato, 2021 [2] and Tapia-Conyer, 2021 [3]^6^ | Mexico | 5–59 years | Vaccination with TIV at 50% coverage | No vaccination | Societal | NA | Cost-saving^7^ | NR | NR |
| LMIC | de Boer, 2018 [1] | Vietnam | All ages | Vaccination with TIV at 15% coverage | No vaccination | Societal | $757.51–1306.31 per QALY | None^5^ | No threshold^5^ | No threshold^5^ |
| **Children**^8^ | | | | | | | | | | |
| UMIC | Giglio, 2012 [4] | Argentina | 6–23 months | Vaccination at 50% coverage; product NR | No vaccination | Healthcare system | $2298.74 per QALY | Cost-effective | $10585.46–31756.39 per QALY | 1–3 times GDP per capita |
| UMIC | Giglio, 2012 [4] | Argentina | 6–36 months | Vaccination at 50% coverage; product NR | No vaccination | Healthcare system | $1441.45 per QALY | Cost-effective | $10585.46–31756.39 per QALY | 1–3 times GDP per capita |
| UMIC | Giglio, 2012 [4] | Argentina | 6 months–5 years | Vaccination at 50% coverage; product NR | No vaccination | Healthcare system | $937.01 per QALY | Cost-effective | $10585.46–31756.39 per QALY | 1–3 times GDP per capita |
| UMIC | Meeyai, 2015 [5] | Thailand | 2–11 years | Vaccination with TIV at 66% coverage | No vaccination | Societal | $5557.72 per DALY | Cost-effective | $12503.30 per DALY | GDP per capita |
| UMIC | Meeyai, 2015 [5] | Thailand | 2–11 years | Vaccination with LAIV at 66% coverage | No vaccination | Societal | $5662.74 per DALY | Cost-effective | $12503.30 per DALY | GDP per capita |
| UMIC | Meeyai, 2015 [5] | Thailand | 2–17 years | Vaccination with LAIV at 66% coverage | No vaccination | Societal | $7186.90 per DALY | Cost-effective | $12503.30 per DALY | GDP per capita |
| UMIC | Meeyai, 2015 [5] | Thailand | 2–5 years | Vaccination with LAIV at 66% coverage | No vaccination | Societal | $3702.23 per DALY | Cost-effective | $12503.30 per DALY | GDP per capita |
| UMIC | Meeyai, 2015 [5] | Thailand | 6–11 years | Vaccination with LAIV at 66% coverage | No vaccination | Societal | $2955.78 per DALY | Cost-effective | $12503.30 per DALY | GDP per capita |
| UMIC | Meeyai, 2015 [5] | Thailand | 12–17 years | Vaccination with LAIV at 66% coverage | No vaccination | Societal | $2301.86 per DALY | Cost-effective | $12503.30 per DALY | GDP per capita |
| UMIC | Zhou, 2014 [6]^9^ | China | 6–59 months | Vaccination at 12% coverage; product NR | No vaccination | Healthcare system | $0 per medically attended case | Cost-effective to cost-saving across seasons | $4153.17–13425.34 per case | GDP per capita (by province) |
| UMIC | Zhou, 2014 [6]^9^ | China | 5–14 years | Vaccination at 11% coverage; product NR | No vaccination | Healthcare system | $48.10 per medically attended case | Cost-effective | $4153.17–13425.34 per case | GDP per capita (by province) |
| UMIC | Kittikraisak, 2017 [7] | Thailand | 6–60 months | Vaccination with TIV at 29–31% coverage | No vaccination | Societal | $668.25– 29,492.86 per QALY | Not cost-effective to cost-effective across seasons | $7209.77–21629.31 per QALY (median threshold) | 1–3 times annual GDP per capita |
| UMIC | Lara, 2018 [8] | Colombia | <5 years | Vaccination with TIV; coverage NR | No vaccination | Healthcare system | NA | Cost-saving | $11761.76–35285.28 per DALY | 1–3 times GDP per capita |
| UMIC | Lara, 2018 [8] | Colombia | <5 years | Vaccination with TIV; coverage NR | No vaccination | Societal | NA | Cost-saving | $11761.76–35285.28 per DALY | 1–3 times GDP per capita |
| UMIC | Falcon-Lezama, 2020 [9] and Tapia-Conyer, 2021 [3]^6^ | Mexico | 5–11 years | Vaccination with TIV at 50% coverage | No vaccination | Societal | NA | Cost-saving^7^ | NR | NR |
| UMIC | Edoka, 2021 [10] | South Africa | 6–59 months | Vaccination with TIV at 2% coverage | No vaccination | Healthcare system | $8507.71 per QALY | Not cost-effective | $3861.98 per QALY | Country-specific threshold^10^ |
| UMIC | Edoka, 2021 [10] | South Africa | 6–59 months | Vaccination with TIV at 2% coverage | No vaccination | Societal | $5982.66 per QALY | Not cost-effective | $3861.98 per QALY | Country-specific threshold^10^ |
| LMIC | Dawa, 2020 [11] | Kenya | 6–23 months | Vaccination with TIV (SH) at 30% coverage | No vaccination | Societal | $850.77–1573.19 per DALY | Not cost-effective | $19.31–990.48 per DALY | 1–51% of GDP per capita |
| LMIC | Dawa, 2020 [11] | Kenya | 2–5 years | Vaccination with TIV (SH) at 35% coverage | No vaccination | Societal | $1073.40–1786.73 per DALY | Not cost-effective | $19.31–990.48 per DALY | 1–51% of GDP per capita |
| LMIC | Dawa, 2020 [11] | Kenya | 6–14 years | Vaccination with TIV (SH) at 40% coverage | No vaccination | Societal | $1048.41–3539.39 per DALY | Not cost-effective | $19.31–990.48 per DALY | 1–51% of GDP per capita |
| LMIC | Dawa, 2020 [11] | Kenya | 6–23 months | Vaccination with TIV (NH) at 30% coverage | No vaccination | Societal | $502.06–2132.04 per DALY | Not cost-effective | $19.31–990.48 per DALY | 1–51% of GDP per capita |
| LMIC | Dawa, 2020 [11] | Kenya | 2–5 years | Vaccination with TIV (NH) at 35% coverage | No vaccination | Societal | $639.50–2122.95 per DALY | Not cost-effective | $19.31–990.48 per DALY | 1–51% of GDP per capita |
| LMIC | Dawa, 2020 [11] | Kenya | 6–14 years | Vaccination with TIV (NH) at 40% coverage | No vaccination | Societal | $1141.56–2525.05 per DALY | Not cost-effective | $19.31–990.48 per DALY | 1–51% of GDP per capita |
| LMIC | Dawa, 2020 [11] | Kenya | 6–23 months | Vaccination with TIV (SH and NH; biannual campaign) at 45% coverage | No vaccination | Societal | $770.12–4663.91 per DALY | Not cost-effective | $19.31–990.48 per DALY | 1–51% of GDP per capita |
| LMIC | Dawa, 2020 [11] | Kenya | 2–5 years | Vaccination with TIV (SH and NH; biannual campaign) at 50% coverage | No vaccination | Societal | $751.95–4640.05 per DALY | Not cost-effective | $19.31–990.48 per DALY | 1–51% of GDP per capita |
| LMIC | Dawa, 2020 [11] | Kenya | 6–14 years | Vaccination with TIV (SH and NH; biannual campaign) at 55% coverage | No vaccination | Societal | $1002.98–5369.29 per DALY | Not cost-effective | $19.31–990.48 per DALY | 1–51% of GDP per capita |
| LMIC | Dawa, 2020 [11] | Kenya | 6–23 months | Vaccination with TIV (SH and NH; year-round) at 60% coverage | No vaccination | Societal | $1302.85–9010.91 per DALY | Not cost-effective | $19.31–990.48 per DALY | 1–51% of GDP per capita |
| LMIC | Dawa, 2020 [11] | Kenya | 2–5 years | Vaccination with TIV (SH and NH; year-round) at 65% coverage | No vaccination | Societal | $1327.84–8970.02 per DALY | Not cost-effective | $19.31–990.48 per DALY | 1–51% of GDP per capita |
| LMIC | Dawa, 2020 [11] | Kenya | 6–14 years | Vaccination with TIV (SH and NH; year-round) at 70% coverage | No vaccination | Societal | $1666.33–7738.73 per DALY | Not cost-effective | $19.31–990.48 per DALY | 1–51% of GDP per capita |
| **Older adults** | | | | | | | | | | |
| UMIC | Meeyai, 2015 [5] | Thailand | ≥60 years | Vaccination with TIV at 66% coverage | Current vaccination program (10% coverage) | Societal | $3612.20 per DALY | Cost-effective | $12503.30 per DALY | GDP per capita |
| UMIC | Chen, 2019 [12] | China | ≥60 years | Vaccination with TIV; coverage NR | No vaccination | Societal | $10313.18 per QALY | Cost-effective | $31238.19 per QALY | 3 times GDP per capita |
| UMIC | Chen, 2019 [12] | China | ≥60 years | Vaccination with QIV; coverage NR | No vaccination | Societal | $28040.63 per QALY | Cost-effective | $31238.19 per QALY | 3 times GDP per capita |
| UMIC | Jiang, 2020 [13] | China | 69 years^11^ | Vaccination with TIV at 27% coverage | No vaccination | Societal | NA | Cost-saving | $32924.00 per QALY | 3 times GDP per capita |
| UMIC | Jiang, 2020 [13] | China | 69 years^11^ | Vaccination with QIV at 27% coverage | No vaccination | Societal | NA | Cost-saving | $32924.00 per QALY | 3 times GDP per capita |
| UMIC | Yang, 2020 [14] | China | ≥60 years | Vaccination with TIV at 30% coverage (fully funded government program) | Current vaccination program (self-paid; 0% coverage) | Societal | $5605.57 per QALY | Cost-effective | $10255.22 per QALY | GDP per capita |
| UMIC | Yan, 2021 [15] | China | ≥60 years | Vaccination with QIV at 48% coverage | No vaccination | Societal | $12133.20 per QALY | Not cost-effective | $11419.14 per QALY | GDP per capita |
| UMIC | Wu, 2022 [16] | China | ≥60 years | Vaccination at 30% coverage; product NR | No vaccination | Societal | NA | Cost-saving | $11419.14 per QALY | GDP per capita |
| UMIC | Edoka, 2021 [10] | South Africa | ≥65 years | Vaccination with TIV at 3% coverage | No vaccination | Healthcare system | $2373.98 per QALY | Cost-effective | $3861.98 per QALY | Country-specific threshold^10^ |
| UMIC | Edoka, 2021 [10] | South Africa | ≥65 years | Vaccination with TIV at 3% coverage | No vaccination | Societal | $2310.37 per QALY | Cost-effective | $3861.98 per QALY | Country-specific threshold^10^ |
| LMIC | Ortega-Sanchez, 2021 [17] | Lao PDR | ≥60 years | Vaccination with TIV at 100% coverage | No vaccination | Societal | $903.24 per life-year saved | Cost-effective | $2783.0–8349.21 per life-year | 1–3 times GDP per capita |
| **Persons with chronic medical conditions** | | | | | | | | | | |
| UMIC | Sribhutorn, 2018 [18] | Thailand | ≤40 years with acute coronary syndrome^12^ | Vaccination with TIV at 100% coverage | No vaccination | Societal | NA | Cost-saving | $5365.64 –16096.91 per life-year | 1–3 times GDP per capita |
| UMIC | Yang, 2019 [19] | China | Persons with diabetes | Vaccination with TIV at 40% coverage | No vaccination | Societal | $1798.51 per QALY | Cost-effective | $9570.18 per QALY | GDP per capita |
| UMIC | Tapia-Conyer, 2021 [3] | Mexico | 12–49 years with UMC^13^ | Vaccination with TIV at 75% coverage | Current vaccination program (9–35% coverage) | Societal | NA | Cost-saving^7^ | NR | NR |
| UMIC | Akin, 2016 [20] | Turkiye | >18 years with diabetes | Vaccination with 20% coverage; product NR | Current program (9% coverage) | Govt. (public payer) | $637.83 per QALY | Cost-effective | $11848.26–35544.78 per QALY | 1–3 times GDP per capita |
| UMIC | Akin, 2016 [20] | Turkiye | >18 years with diabetes | Vaccination with 20% coverage; product NR | Current program (9% coverage) | Societal | $35.25 per QALY | Cost-effective | $11848.26–35544.78 per QALY | 1–3 times GDP per capita |
| UMIC | Choosakulchart, 2013 [21] | Thailand | ≥60 years with coronary heart disease^14^ | Vaccination with product and coverage NR | No vaccination | Societal | $1386.98 per QALY | Cost-effective | $4101.92 per QALY | Details not specified^15^ |
| UMIC | Edoka, 2021 [10] | South Africa | Persons living with HIV/AIDS | Vaccination with TIV at 5.51% coverage | No vaccination | Healthcare system | $1986.65 per QALY | Cost-effective | $3861.98 per QALY | Country-specific threshold^10^ |
| UMIC | Edoka, 2021 [10] | South Africa | Persons living with HIV/AIDS | Vaccination with TIV at 5.51% coverage | No vaccination | Societal | NA | Cost-saving | $3861.98 per QALY | Country-specific threshold^10^ |
| UMIC | Edoka, 2021 [10] | South Africa | Persons with other UMC | Vaccination with TIV at 3.14% coverage | No vaccination | Healthcare system | $2973.72 per QALY | Cost-effective | $3861.98 per QALY | Country-specific threshold^10^ |
| UMIC | Edoka, 2021 [10] | South Africa | Persons with other UMC | Vaccination with TIV at 3.14% coverage | No vaccination | Societal | NA | Cost-saving | $3861.98 per QALY | Country-specific threshold^10^ |
| **Pregnant persons (including infants <6 months)** | | | | | | | | | | |
| UMIC | Biggerstaff, 2019 [22] | South Africa | NA | Vaccination prioritizing persons with HIV (70% coverage in HIV+ and 44% coverage in HIV-); product NR | No vaccination | Societal | $5550.81 per QALY | Potentially cost-effective^16^ | $6776.03 per QALY | GDP per capita |
| UMIC | Biggerstaff, 2019 [22] | South Africa | NA | Vaccination with 50% coverage; product NR | No vaccination | Societal | $7012.79 per QALY | Potentially cost-effective^16^ | $6776.03 per QALY | GDP per capita |
| UMIC | Edoka, 2021 [10] | South Africa | NA | Vaccination with TIV at 48.9% coverage | No vaccination | Healthcare system | $2283.11 per QALY | Cost-effective | $3861.98 per QALY | Country-specific threshold^10^ |
| UMIC | Edoka, 2021 [10] | South Africa | NA | Vaccination with TIV at 48.9% coverage | No vaccination | Societal | NA | Cost-saving | $3861.98 per QALY | Country-specific threshold^10^ |
| LMIC | Ortega-Sanchez, 2021 [17] | Lao PDR | NA | Vaccination with TIV at 100% coverage | No vaccination | Societal | $5797.15 per life-year saved | Cost-effective | $2783.0–8349.21 per life-year | 1–3 times GDP per capita |
| LIC | Orenstein, 2017 [23] | Mali | NA | Vaccination with TIV; coverage NR^17^ | No vaccination | Societal | $1033.76 per DALY | Cost-effective^18^ | $862.47 per DALY | GDP per capita |
| **Healthcare workers** | | | | | | | | | | |
| UMIC | Tohiar, 2022 [24] | Malaysia | NA | Vaccination with QIV at 63% coverage | No vaccination | Employer | NA | Cost-saving^7^ | NR | NR |
| LMIC | Ortega-Sanchez, 2021 [17] | Lao PDR | NA | Vaccination with TIV at 100% coverage | No vaccination | Societal | NA | Cost-saving | $2783.0–8349.21 per life-year | 1–3 times GDP per capita |
| LMIC | Kyi-Kokarieva, 2021 [25] | Ukraine | NA | Vaccination at 70% coverage; product NR | No vaccination | Societal | NA | Cost-saving^7^ | NR | NR |
| **Persons in congregate living settings** | | | | | | | | | | |
| UMIC | Suphanchaimat, 2020 [26] | Thailand | Incarcerated persons | Vaccination with TIV at 10% coverage^19^ | No vaccination | Govt. | $2108.55 per DALY | Cost-effective | NR | NR |

Abbreviations: DALY, disability-adjusted life year; ICER, incremental cost-effectiveness ratio; Govt, government; HIV, human immunodeficiency virus; LAIV, live attenuated influenza vaccine; LIC, low-income country; LMIC, lower-middle income country; NA, not applicable; NR, not reported; QALY, quality-adjusted life year; QIV, quadrivalent influenza vaccine; TIV, trivalent influenza vaccine; UMIC, upper-middle income country; US$, US Dollars; WTP, willingness-to-pay.

^1^Cost-effectiveness, cost-utility, and cost-benefit analyses were eligible for inclusion if they included a comparison of influenza vaccination vs. either no vaccination or modifications to current vaccination program. Studies that only compared the cost-effectiveness of different influenza vaccine products were not included.

^2^Data for each base-scenario intervention or each perspective assessed are presented in individual rows. Sensitivity analyses are not presented. Vaccine coverage was rounded to the nearest integer.

^3^Calculated or converted value; not presented in source publication. Ranges represent annual seasonal estimates or varying illness attack rate.

^4^Interpretation per source publication. Interpretations of highly cost-effective and cost-effective were both combined as “cost-effective.”

^5^The study authors intentionally did not specify a cost-effectiveness threshold or interpretation because a country-specific threshold was not available. Instead, cost-effectiveness acceptability curves were presented over a range of willingness-to-pay thresholds.

^6^Similar data sources and analysis methods used in both publications; these were counted collectively as one study for Figure 3.

^7^Interpretation of net costs of vaccination (including illness averted) vs. no vaccination.

^8^Although SAGE recommendations specifically reference children aged <5 years [27], publications with data for children aged <18 years were included.

^9^This study also modeled alternative strategies to increase vaccination rates.

^10^This study used a cost-effectiveness threshold for South Africa that reflects the health opportunity cost of health spending.

^11^The age of the hypothetical cohort was based on the mean age of the target population in China (69 years).

^12^Age groups of ≥50 years and ≥60 years were also modeled; only results for ≥40 years are shown as this was inclusive of all other groups. All scenarios were cost-effective.

^13^Medical conditions included diabetes, high blood pressure, morbid obesity, chronic renal failure, asthma, and pregnancy.

^14^Included patients with angina and cardiac arrest/myocardial infarction.

^15^A country-specific threshold of 100,000 Thai Baht was used (rationale not reported).

^16^The 90% uncertainty intervals for the ICER overlapped the cost-effectiveness threshold.

^17^Additional scenarios adjusted for poor access to care and increased severity of disease; all scenarios were cost-effective.

^18^Results were interpreted as cost-effective when the cost per pregnant woman vaccinated was $1.00 or less.

^19^Additional scenarios modeled higher coverage of 30% and 100%; all scenarios were cost-effective.

**References**

1. de Boer PT, Kelso JK, Halder N, Nguyen TP, Moyes J, Cohen C, et al. The cost-effectiveness of trivalent and quadrivalent influenza vaccination in communities in South Africa, Vietnam and Australia. Vaccine. 2018;36(7):997-1007. doi: <https://dx.doi.org/10.1016/j.vaccine.2017.12.073>.

2. Betancourt-Cravioto M, Falcon-Lezama JA, Saucedo-Martinez R, Alfaro-Cortes MM, Tapia-Conyer R. Public Health and Economic Benefits of Influenza Vaccination of the Population Aged 50 to 59 Years without Risk Factors for Influenza Complications in Mexico: A Cross-Sectional Epidemiological Study. Vaccines (Basel). 2021;9(3):24. doi: <https://dx.doi.org/10.3390/vaccines9030188>.

3. Tapia-Conyer R, Betancourt-Cravioto M, Montoya A, Falcon-Lezama JA, Alfaro-Cortes MM, Saucedo-Martinez R. A Call for a Reform of the Influenza Immunization Program in Mexico: Epidemiologic and Economic Evidence for Decision Making. Vaccines (Basel). 2021;9(3):19. doi: <https://dx.doi.org/10.3390/vaccines9030286>.

4. Giglio N, Gentile A, Lees L, Micone P, Armoni J, Reygrobellet C, et al. Public health and economic benefits of new pediatric influenza vaccination programs in Argentina. Hum Vaccin Immunother. 2012;8(3):312-22. doi: <https://dx.doi.org/10.4161/hv.18569>.

5. Meeyai A, Praditsitthikorn N, Kotirum S, Kulpeng W, Putthasri W, Cooper BS, et al. Seasonal influenza vaccination for children in Thailand: a cost-effectiveness analysis. PLoS Med. 2015;12(5):e1001829. doi: <https://dx.doi.org/10.1371/journal.pmed.1001829>.

6. Zhou L, Situ S, Feng Z, Atkins CY, Fung IC, Xu Z, et al. Cost-effectiveness of alternative strategies for annual influenza vaccination among children aged 6 months to 14 years in four provinces in China. PLoS ONE. 2014;9(1):e87590. doi: <https://dx.doi.org/10.1371/journal.pone.0087590>.

7. Kittikraisak W, Suntarattiwong P, Ditsungnoen D, Pallas SE, Abimbola TO, Klungthong C, et al. Cost-effectiveness of inactivated seasonal influenza vaccination in a cohort of Thai children 60 months of age. PLoS One. 2017;12(8). doi: <http://dx.doi.org/10.1371/journal.pone.0183391>.

8. Lara C, De Graeve D, Franco F. Cost-Effectiveness Analysis of Pneumococcal and Influenza Vaccines Administered to Children Less Than 5 Years of Age in a Low-Income District of Bogota, Colombia. Value Health Reg Issues. 2018;17:21-31. doi: <https://dx.doi.org/10.1016/j.vhri.2018.01.001>.

9. Falcon-Lezama JA, Saucedo-Martinez R, Betancourt-Cravioto M, Alfaro-Cortes MM, Bahena-Gonzalez RI, Tapia-Conyer R. Influenza in the school-aged population in Mexico: burden of disease and cost-effectiveness of vaccination in children. BMC Infect Dis. 2020;20(1):240. doi: <https://dx.doi.org/10.1186/s12879-020-4948-5>.

10. Edoka I, Kohli-Lynch C, Fraser H, Hofman K, Tempia S, McMorrow M, et al. A cost-effectiveness analysis of South Africa's seasonal influenza vaccination programme. Vaccine. 2021;39(2):412-22. doi: <https://dx.doi.org/10.1016/j.vaccine.2020.11.028>.

11. Dawa J, Emukule GO, Barasa E, Widdowson MA, Anzala O, van Leeuwen E, et al. Seasonal influenza vaccination in Kenya: an economic evaluation using dynamic transmission modelling. BMC Med. 2020;18(1):223. doi: <https://dx.doi.org/10.1186/s12916-020-01687-7>.

12. Chen C, Liu GE, Wang MJ, Gao TF, Jia HP, Yang H, et al. Cost-effective analysis of seasonal influenza vaccine in elderly Chinese population [in Chinese]. Zhonghua Yu Fang Yi Xue Za Zhi. 2019;53(10):993-9. doi: <https://dx.doi.org/10.3760/cma.j.issn.0253-9624.2019.10.008>.

13. Jiang M, Li P, Wang W, Zhao M, Atif N, Zhu S, et al. Cost-effectiveness of quadrivalent versus trivalent influenza vaccine for elderly population in China. Vaccine. 2020;38(5):1057-64. doi: <https://dx.doi.org/10.1016/j.vaccine.2019.11.045>.

14. Yang J, Atkins KE, Feng L, Baguelin M, Wu P, Yan H, et al. Cost-effectiveness of introducing national seasonal influenza vaccination for adults aged 60 years and above in mainland China: a modelling analysis. BMC Med. 2020;18(1):90. doi: <https://dx.doi.org/10.1186/s12916-020-01545-6>.

15. Yan H, Yang J, Chen Z, Gong H, Zhong G, Yu H. Cost-effectiveness analysis of quadrivalent influenza vaccination for older adults aged 60 and above in mainland China [in Chinese]. Zhonghua Yi Xue Za Zhi. 2021;101(30):2405-12. doi: <https://dx.doi.org/10.3760/cma.j.cn112137-21210123-00224>.

16. Wu XL, Ye ZJ, Xie F, Huang DF, Kong TJ, Feng SX, et al. Based on a Markov model, cost-effectiveness analysis of influenza vaccination among people aged 60 years and older in Shenzhen [in Chinese]. Zhonghua Liu Xing Bing Xue Za Zhi. 2022;43(7):1140-6. doi: <https://dx.doi.org/10.3760/cma.j.cn112338-20211221-01005>.

17. Ortega-Sanchez IR, Mott JA, Kittikraisak W, Khanthamaly V, McCarron M, Keokhonenang S, et al. Cost-effectiveness of seasonal influenza vaccination in pregnant women, healthcare workers and adults >= 60 years of age in Lao People's Democratic Republic. Vaccine. 2021;39(52):7633-45. doi: <https://dx.doi.org/10.1016/j.vaccine.2021.11.011>.

18. Sribhutorn A, Phrommintikul A, Wongcharoen W, Chaikledkaew U, Eakanunkul S, Sukonthasarn A. Influenza vaccination in acute coronary syndromes patients in Thailand: the cost-effectiveness analysis of the prevention for cardiovascular events and pneumonia. J Geriatr Cardiol. 2018;15(6):413-21. doi: <https://dx.doi.org/10.11909/j.issn.1671-5411.2018.06.008>.

19. Yang J, Yan H, Feng LZ, Yu HJ. Cost-effectiveness of potential government fully-funded influenza vaccination in population with diabetes in China [in Chinese]. Zhonghua Yu Fang Yi Xue Za Zhi. 2019;53(10):1000-6. doi: 10.3760/cma.j.issn.0253-9624.2019.10.009.

20. Akin L, Macabeo B, Caliskan Z, Altinel S, Satman I. Cost-Effectiveness of Increasing Influenza Vaccination Coverage in Adults with Type 2 Diabetes in Turkey. PLoS ONE. 2016;11(6):e0157657. doi: <https://dx.doi.org/10.1371/journal.pone.0157657>.

21. Choosakulchart P, Kittisopee T, Takdhada S, Lubell Y, Robinson J. Cost-utility evaluation of influenza vaccination in patients with existing coronary heart diseases in Thailand. Asian Biomedicine. 2013;7(3):425-35. doi: <http://dx.doi.org/10.5372/1905-7415.0703.196>.

22. Biggerstaff M, Cohen C, Reed C, Tempia S, McMorrow ML, Walaza S, et al. A cost-effectiveness analysis of antenatal influenza vaccination among HIV-infected and HIV-uninfected pregnant women in South Africa. Vaccine. 2019;37(46):6874-84. doi: <https://dx.doi.org/10.1016/j.vaccine.2019.09.059>.

23. Orenstein EW, Orenstein LA, Diarra K, Djiteye M, Sidibe D, Haidara FC, et al. Cost-effectiveness of maternal influenza immunization in Bamako, Mali: A decision analysis. PLoS ONE. 2017;12(2):e0171499. doi: <https://dx.doi.org/10.1371/journal.pone.0171499>.

24. Tohiar MAH, Jaafar S, Aizuddin AN, Leong TK, Abdul Rahim AS. Workplace influenza vaccination in private hospital setting: a cost-benefit analysis. Ann Occup Environ Med. 2022;34:e3. doi: <https://dx.doi.org/10.35371/aoem.2022.34.e3>.

25. Kyi-Kokarieva VG, Padalkо LI, Kriachkova LV. Socio-economic substantiation of expediency of seasonal influenza vaccine prophylaxis among medical workers. Medicni Perspektivi. 2021;26(4):205-12. doi: 10.26641/2307-0404.2021.4.248235.

26. Suphanchaimat R, Doung-Ngern P, Ploddi K, Suthachana S, Phaiyarom M, Pachanee K, et al. Cost Effectiveness and Budget Impact Analyses of Influenza Vaccination for Prisoners in Thailand: An Application of System Dynamic Modelling. Int J Environ Res Public Health. 2020;17(4):14. doi: <https://dx.doi.org/10.3390/ijerph17041247>.

27. World Health Organization. Vaccines against influenza: WHO position paper—May 2022. Wkly Epidemiol Rec. 2022;19:185-208.
